# Pharmacological hypogonadism impairs molecular transducers of exercise‐induced muscle growth in humans

**DOI:** 10.1002/jcsm.12843

**Published:** 2022-03-01

**Authors:** Nima Gharahdaghi, Supreeth Rudrappa, Matthew S. Brook, Wesam Farrash, Iskandar Idris, Muhammad Hariz Abdul Aziz, Fawzi Kadi, Konstantinos Papaioannou, Bethan E. Phillips, Tanvir Sian, Philip J. Herrod, Daniel J. Wilkinson, Nathaniel J. Szewczyk, Kenneth Smith, Philip J. Atherton

**Affiliations:** ^1^ MRC‐Verus Arthritis Centre for Musculoskeletal Ageing Research and Nottingham NIHR BRC, School of Medicine University of Nottingham Derby UK; ^2^ Laboratory Medicine Department, College of Applied Medical Sciences Umm Al-Qura University Makkah Saudi Arabia; ^3^ Division of Sports Sciences, School of Health and Medical Sciences Örebro University Örebro Sweden

**Keywords:** Testosterone, Exercise training, Muscle protein synthesis, Hypertrophy

## Abstract

**Background:**

The relative role of skeletal muscle mechano‐transduction in comparison with systemic hormones, such as testosterone (T), in regulating hypertrophic responses to exercise is contentious. We investigated the mechanistic effects of chemical endogenous T depletion adjuvant to 6 weeks of resistance exercise training (RET) on muscle mass, function, myogenic regulatory factors, and muscle anabolic signalling in younger men.

**Methods:**

Non‐hypogonadal men (*n* = 16; 18–30 years) were randomized in a double‐blinded fashion to receive placebo (P, saline *n* = 8) or the GnRH analogue, Goserelin [Zoladex (Z), 3.6 mg, *n* = 8], injections, before 6 weeks of supervised whole‐body RET. Participants underwent dual‐energy X‐ray absorptiometry (DXA), ultrasound of m. vastus lateralis (VL), and VL biopsies for assessment of cumulative muscle protein synthesis (MPS), myogenic gene expression, and anabolic signalling pathway responses.

**Results:**

Zoladex suppressed endogenous T to within the hypogonadal range and was well tolerated; suppression was associated with blunted fat free mass [Z: 55.4 ± 2.8 to 55.8 ± 3.1 kg, *P =* 0.61 vs. P: 55.9 ± 1.7 to 57.4 ± 1.7 kg, *P =* 0.006, effect size (ES) = 0.31], composite strength (Z: 40 ± 2.3% vs. P: 49.8 ± 3.3%, *P =* 0.03, ES = 1.4), and muscle thickness (Z: 2.7 ± 0.4 to 2.69 ± 0.36 cm, *P* > 0.99 vs. P: 2.74 ± 0.32 to 2.91 ± 0.32 cm, *P* < 0.0001, ES = 0.48) gains. Hypogonadism attenuated molecular transducers of muscle growth related to T metabolism (e.g. *androgen receptor*: Z: 1.2 fold, *P* > 0.99 vs. P: 1.9 fold, *P* < 0.0001, ES = 0.85), anabolism/myogenesis (e.g. *IGF‐1Ea*: Z: 1.9 fold, *P =* 0.5 vs. P: 3.3 fold, *P =* 0.0005, ES = 0.72; *IGF‐1Ec*: Z: 2 fold, *P* > 0.99 vs. P: 4.7 fold, *P =* 0.0005, ES = 0.68; myogenin: Z: 1.3 fold, *P* > 0.99 vs. P: 2.7 fold, *P =* 0.002, ES = 0.72), RNA/DNA (Z: 0.47 ± 0.03 to 0.53 ± 0.03, *P =* 0.31 vs. P: 0.50 ± 0.01 to 0.64 ± 0.04, *P =* 0.003, ES = 0.72), and RNA/ASP (Z: 5.8 ± 0.4 to 6.8 ± 0.5, *P* > 0.99 vs. P: 6.5 ± 0.2 to 8.9 ± 1.1, *P =* 0.008, ES = 0.63) ratios, as well as acute RET‐induced phosphorylation of growth signalling proteins (e.g. AKT^ser473^: Z: 2.74 ± 0.6, *P =* 0.2 vs. P: 5.5 ± 1.1 fold change, *P* < 0.001, ES = 0.54 and mTORC1^ser2448^: Z: 1.9 ± 0.8, *P* > 0.99 vs. P: 3.6 ± 1 fold change, *P =* 0.002, ES = 0.53). Both MPS (Z: 1.45 ± 0.11 to 1.50 ± 0.06%·day^−1^, *P =* 0.99 vs. P: 1.5 ± 0.12 to 2.0 ± 0.15%·day^−1^, *P =* 0.01, ES = 0.97) and (extrapolated) muscle protein breakdown (Z: 93.16 ± 7.8 vs. P: 129.1 ± 13.8 g·day^−1^, *P =* 0.04, ES = 0.92) were reduced with hypogonadism result in lower net protein turnover (3.9 ± 1.1 vs. 1.2 ± 1.1 g·day^−1^, *P =* 0.04, ES = 0.95).

**Conclusions:**

We conclude that endogenous T sufficiency has a central role in the up‐regulation of molecular transducers of RET‐induced muscle hypertrophy in humans that cannot be overcome by muscle mechano‐transduction alone.

## Introduction

Skeletal muscle functions as a motor for locomotion and regulator of whole‐body metabolism.^1^ Catabolic conditions in skeletal muscle, for example, ageing, disuse, cachexia, and denervation, lead to muscle atrophy accompanied by disability, loss of independence, morbidity, and mortality.^2^ The regulatory factors that maintain muscle mass, both structurally and metabolically, dictate the balance between muscle protein synthesis (MPS) and breakdown (MPB).^3^ In turn, muscle proteostasis is under the influence of a myriad of factors such as nutrition, hormonal milieu, injury, disease, and exercise.^3^ In terms of the latter, a major focus has—and remains—the control of muscle mass and the use of resistance exercise training (RET) as a countermeasure for muscle atrophy. Nonetheless, the central mechanisms of successful muscle maintenance and growth remain contentious.

The two major routes to muscle growth are (i) hormonal/humoral and (ii) mechano‐pathways. Mechano‐transduction describes the conversion of physically induced factors (e.g. stretch/active‐contraction) into chemical messengers to aid the cell to mount an appropriate response via modulation of target transcription and mRNA translation. One of the most widely recognized players in controlling MPS (and by extension, mass) is the mechanistic target of rapamycin (mTOR) pathway, indicating the essentiality of post‐translational signalling pathways in relation to MPS.^4^ Reflecting this, mTOR complex 1 (mTORC1) co‐ordinates cellular remodelling processes of growth, differentiation, autophagy, and survival via its substrates [e.g. ribosomal S6 kinase 1 (S6K1) and 4E‐binding protein 1 (4EBP1)].^5^ mTORC1 activation is triggered by the IGF‐1–PI3K–Akt signalling pathway where insulin‐like growth factor 1 (IGF‐1) binds to its receptor and phosphorylates phosphatidyl inositol 3‐kinase (PI3K), which in turn activates protein kinase B (Akt).^6^ mTORC1 may also be activated following mechanical overload but independently of the IGF‐1–PI3K–Akt pathway, without Akt phosphorylation^7^ or with reduced activation after RE in the fasted state, despite downstream activation of mTORC1.^8^ mTORC1 activation at the early phase of mechanical overload via mitogen activated kinase/extracellular signal‐regulated kinase (MEK/ERK)/tuberous sclerosis protein 2 (TSC2) signalling is also associated with delayed increases in total RNA content (~5 days) and muscle hypertrophy in rats.^7^ Muscle growth is also regulated by temporal transcriptional remodelling during RET.^9^ However, the upstream drivers, that is, the stimulatory molecular transducers of muscle growth, are a myriad, and the integrated effects of hormones (e.g. androgens) and mechano‐transduction processes remain to be fully elucidated.^10^

Hormones, androgens specifically, are established regulators of protein turnover, through genomic (transcriptional capacity) and non‐genomic (translational efficiency) pathways.^10^ Exemplifying this, androgen deprivation therapy was associated with a decrease in fat free mass (FFM) and increase in fat mass.^11^ We also showed that older men with lower baseline levels of endogenous testosterone (T) exhibited blunted adaptation to RET, which could be overcome with exogenous T administration,^12^, ^13^ thus suggesting a major role for T in the regulation of molecular transducers of human muscle growth. Similarly, irrespective of age, reducing endogenous T levels to a castration range for 12 weeks blunted FFM and strength gains in young men.^14^ However, other work points to a limited role of T in mRNA translation and muscle growth, and in relation to RE.^15^, ^16^ For instance, post‐RE elevations in T were not associated with increases in FFM, fibre cross‐sectional area (CSA), or strength after 12 weeks of RET,^16^ while a ‘high’ versus ‘low’ hormone environment (induced by working distinct muscle bulk) did not enhance hypertrophy or strength in young men.^15^ Finally, RE‐induced increases in endogenous T did not enhance anabolic signalling or acute MPS responses.^17^ It is extraordinary that a major area of contention remains the role of endogenous T in exercise‐induced muscle growth, for example, in comparison with non‐centrally mediated effectors (mechano‐transduction). To resolve this, we studied the impacts of RET under pharmaceutical hypogonadism (NB. not, depletion) in younger men, while determining endpoints relating to muscle mass, function, myogenic regulation, anabolic signalling, and protein turnover. Our study design permitted determination of the impact of hypogonadism, with a translational relevance.

## Materials and methods

### Study overview and participants details

This study was approved by The University of Nottingham Faculty of Medicine and Health Sciences Research Ethics Committee (G11082015 SoMS MSGEM), was conducted according to the Declaration of Helsinki, and was pre‐registered at clinicaltrials.gov (NCT02152839). Before entry into the study, participants provided written informed consent to participate after all procedures and risks were explained to them. All participants were screened by medical questionnaire, physical examination, routine blood chemistry, and a resting electrocardiogram, and those who presented with metabolic, respiratory, or cardiovascular disorders or who were prescribed chronic medication (e.g. beta‐adrenergic blocking agents, statins, and anti‐inflammatory drugs) or any other medication that could influence T metabolism were excluded.

**Figure 1:**
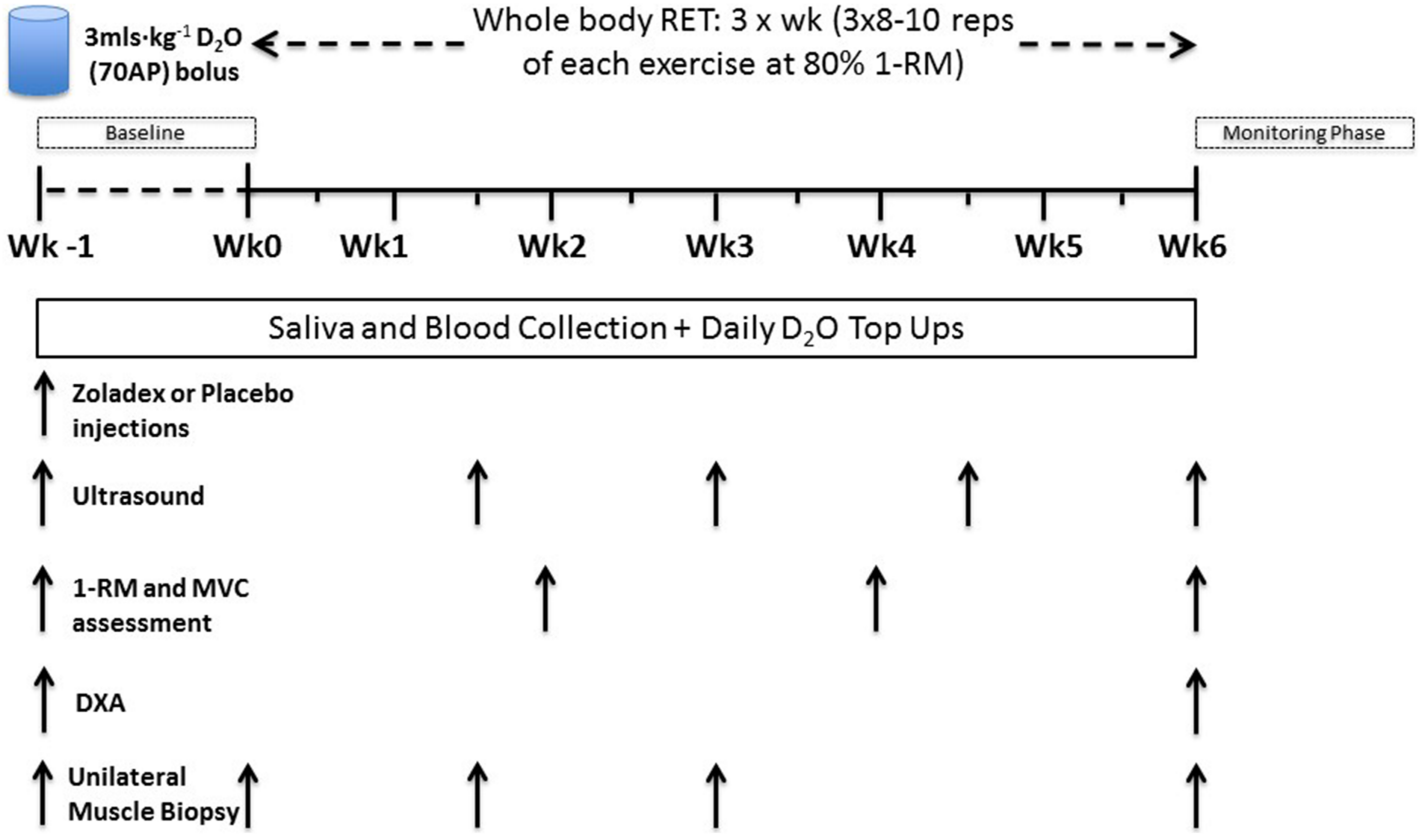
Detailed schematic of the study protocol.

Following baseline measurements of maximal voluntary contraction (MVC) and 1‐repetition maximum (1‐RM; on separate days), regardless of group assignment, all participants were further characterized at baseline. This involved collection of a fasting blood sample, muscle ultrasound (Mylab 70; Esaote Biomedica, Italy) of the m. vastus lateralis muscle (VL), and a dual‐energy X‐ray absorptiometry (DXA; Lunar Prodigy II, GE Medical Systems, Little Chalfont, UK) scan. Finally, a unilateral muscle biopsy was taken under rested conditions from the VL. In order to assess rates of MPS, a basal saliva sample was collected before the muscle biopsy, and the first dose of D_2_O as a bolus of 3 mL·kg^−1^ body weight was consumed by participants after the biopsy. The initial priming dose of D_2_O was followed by daily small‐volume ‘top‐ups’ of ~20 mL (calculated from measures of each individual's body water pool turnover). Finally, a subcutaneous injection of Zoladex or saline placebo (P) was administered by an unblinded clinical research fellow; in order to identify any potential side effects of the Zoladex administration, they were not involved in any other aspect of the study. The fully supervised RET protocol then commenced and continued for the next 6 weeks. Additional VL biopsies (60 min after bouts of RE to obtain temporal acute effects of RE across RET) and other tests/samples took place intermittently during these 6 weeks. A detailed schematic of the study protocol is depicted in *Figure* 1. All muscle samples were collected under sterile conditions, using the conchotome biopsy technique with 1% w·v^−1^ lidocaine as local anaesthetic. Any fat tissue and connective tissue were rapidly dissected out, and muscle was washed in ice‐cold phosphate‐buffered saline (PBS) and frozen in liquid nitrogen or liquid‐nitrogen cooled isopentane, before storage at −80°C. All participants involved in the study were monitored throughout the study, by the CRF, for any negative side effects of Z. No adverse events were reported during or after completion of the study. Physiological characteristics of participants are shown in *Table* 1.

**Table 1:** Participants' physiological characteristics before and after resistance exercise training

	Z (*n* = 8)		P (*n* = 8)	
	Baseline	Week 6	Baseline	Week 6
Age (years)	22.2 (0.8)	––	21.1 (1.2)	––
Height (m)	1.7 (0.1)	––	1.8 (0.1)	––
Weight (kg)	72.9 (4.1)	74.1 (4.3)	75.7 (4.4)	76.7 (4.3)
BMI (kg·m^−2^)	23.1 (1.1)	23.4 (1.1)	22.9 (1.4)	23.2 (1.4)

Values are means (SEM). BMI, body mass index.

### Physiological and functional measurements

#### Resistance training procedures and strength measurement

Participants in both groups performed the same whole‐body RET including leg extension, leg press, leg curl, lat pull‐down, shoulder press, and bench press (all 3 sets of 8–10 reps at 80% 1‐RM)^13^ three times per week on non‐consecutive days for 6 weeks as a well‐established model of hypertrophy.^18^ Individuals' 1‐RM was re‐assessed every 2 weeks before a training session to maintain intensity with progression.^13^ An isokinetic dynamometer (Isocom; Isokinetic Technologies, Eurokinetics, UK) was used to assess isometric knee extensor torque during MVC using three knee joint angles (60°, 70°, and 80°), with full extension corresponding to 0°. Each individual muscle contraction lasted 4 s, with 30 s of rest between contractions, and 90 s between different knee joint angle assessments. In addition, specific strength (total strength·FFM^−1^) was calculated as total strength divided by total FFM.

#### Muscle architecture by ultrasound and dual‐energy X‐ray absorptiometry‐derived fat free mass

Every 10 days and immediately before a training session (e.g. ∼72 h after last training session), B‐mode ultrasonography (Mylab 70, Esaote Biomedica) with a 100 mm, 10–15 MHz, linear array probe was used for quantification of myo‐architecture. Images were obtained at 50% of the VL length and the mid‐sagittal line while the participant was lying supine on a couch.^13^ To assess fascicle length (Lf), the transducer was aligned with the fascicles to facilitate optimal image capture of the fascicles. The intersection between fascicles and the deep tendon aponeurosis and the perpendicular distance between the superficial and the deep tendon aponeurosis were used to assess pennation angle (PA) and muscle thickness (MT), respectively. Finally, extended‐field‐of‐view ultrasonography (EFOV) was used to quantify the CSA of the quadriceps. ImageJ software (ImageJ 1.51h) was used to analyse the ultrasound images.

Before and after the study (i.e. ∼72 h after last training session), DXA (64752, GE Medical Systems‐Lunar Prodigy, US) was used to determine total FFM, leg FFM, total fat percentage (TFP), total fat mass (TFM), FFM index [FFM divided by height squared (FFM·height^−2^)], and appendicular FFM (FFM of arms and legs in kilograms divided by square of height in metres). Participants were asked to attend having fasted overnight and not performed any heavy physical activity 24 h prior to scanning. For the DXA scan, participants wore loose comfortable clothing with no metal or plastic zippers, buttons, or snaps. Prior to use on participants, a QA block phantom was used to calibrate the system, to ensure optimal measurement. In addition, spine phantoms were run monthly to ensure the reproducibility and accuracy of the system over time.

### Metabolic and biochemical measurements

#### Testosterone enzyme‐linked immunosorbent assay

Venous blood samples were collected into EDTA‐coated tubes intermittently during the study, that is, before injections and prior to individual RE sessions in the morning to measure fluctuations in total T concentrations. Blood samples were immediately cold centrifuged at 1750 *g*, with resulting plasma fractions aliquoted and frozen at −80°C until further analysis. An enzyme‐linked immunosorbent assay (ELISA; RE52151, IBL, Germany) competitive technique was used to assess the abundance of total T in the plasma of all participants. The intra‐assay coefficient of variation was <5%, the assay sensitivity was 70 ng·dL^−1^, and the detection range was 20–1154 ng·dL^−1^.

#### Muscle immunohistochemistry

Serial 5‐μm‐thick VL muscle cross‐sections were cut at −20°C using a cryostat (Leica, CM 1850, Germany), mounted on glass slides, and air‐dried at room temperature. Determination of fibre type‐specific CSA, myonuclear, and satellite cell number was performed using monoclonal antibodies against slow (BA‐F8) and fast myosin (SC‐71), laminin (D18), and Pax‐7 (Developmental Studies Hybridoma Bank). Visualization of the primary antibodies was achieved by incubation with Alexa Fluor 488 for BA‐F8 and D18 and with Alexa Fluor 568 for SC‐71 goat‐anti‐mouse secondary antibodies (Invitrogen A/S) *in situ*. Visualization of Pax‐7 was performed after incubation with DAB. Myonuclei were stained with DAPI. Muscle sections were then mounted with Molecular Probes Prolong Gold antifade reagent (Invitrogen A/S). Three major fibre types (I, IIA, and IIX) were determined as previously described.^19^ Fibre area was measured using Sigma Scan Pro 5 software.

#### Body water and myofibrillar protein‐bound alanine enrichment to determine muscle protein synthesis

To determine the exact volume of D_2_O to be consumed for daily ‘top‐ups’, each participant provided saliva for the first 3 days after initial D_2_O consumption. These were processed to determine each participant's body water decay rate, and from this, the amount of D_2_O needed to maintain a steady state over the study period could be calculated. Individuals were then provided with stocks of daily D_2_O ‘top‐ups’ (∼10% initial bolus dose) with thrice weekly saliva collection for rest of the study period. Wilkinson *et al*. previously described how body water and muscle protein enrichment were analysed.^20^ Briefly, 80–90 μL of saliva was heated in inverted 2 mL of autosampler vials for 4 h at 90–100°C to evaporate the body water. The vials were then cooled on ice, and the condensate, that is, body water, was transferred to a clean autosampler vial for injection. A high‐temperature conversion elemental analyser (Thermo Finnigan, Thermo Scientific, Hemel Hempstead, UK) connected to an isotope ratio mass spectrometer (Delta V advantage, Thermo Scientific) was employed to measure deuterium labelling in saliva (0.1 μL). To assess protein bound alanine muscle fraction enrichment, ~40 mg of muscle was homogenized in ice‐cold homogenization buffer (Supporting Information, *Table* S2) to isolate myofibrillar proteins.^12^, ^13^ Briefly, 10 min of rotary mixing was followed by centrifugation at 11 000 *g* for 15 min at 4°C, the supernatant (sarcoplasmic fraction) was then collected for immunoblotting, and the pellet was re‐suspended in 500 μL of mitochondrial extraction buffer (MEB) (*Table* S2), then homogenized by Dounce, and centrifuged at 1000 g for 5 min at 4°C. Insoluble collagen was separated following centrifugation from myofibrillar proteins that were solubilized in 750 μL of 0.3 N NaOH and subsequently precipitated using 1 Ϻ perchloric acid (PCA) then pelleted by centrifugation. Following overnight hydrolysis at 110°C in a 0.1 Ϻ HCl and Dowex H^+^ resin slurry, the amino acids were eluted with 2 Ϻ NH_4_OH and dried‐down. Dried samples were suspended in 60 μL of distilled water, 32 μL of methanol, and 10 μL of pyridine and 8 μL of methyl chloroformate with intermittent vortex mixing. The n‐methoxycarbonyl methyl esters of the amino acids were then extracted after adding 100 μL of chloroform. A molecular sieve was added to remove water for ∼20 s before being transferred to autosampler vials; incorporation of deuterium into the protein bound alanine was determined by gas chromatography‐pyrolysis‐isotope ratio mass spectrometry (Delta V Advantage, Thermo, Hemel Hempstead, UK).^12^, ^13^

##### Calculation of synthetic fractional rate

Myofibrillar MPS was calculated from the deuterium enrichment [i.e. atom per cent excess (APE)] of alanine in myofibrillar proteins, using the body water enrichment (APE, corrected for the mean number of deuterium moieties incorporated per alanine; 3.7, and the dilution from the total number of hydrogens in the derivative, i.e. 11) as the precursor labelling between subsequent biopsies. The fractional synthetic rate (FSR) was calculated as follows:

$$\mathrm{FSR}\left(\%\cdot {\mathrm{day}}^{-1}\right)=-\text{In}\left(\frac{1-\left[\frac{\left({\mathrm{APE}}_{\mathrm{Ala}}\right.}{{\mathrm{APE}}_{P}}\right]}{t}\right),$$

where APE_Ala_ is deuterium enrichment of protein‐bound alanine, APE_P_ is mean precursor enrichment of the body water over the period, and *t* is the time between biopsies.

Absolute synthetic rate (ASR) was estimated as

$$\mathrm{ASR}\left(g\cdot {\mathrm{day}}^{-1}\right)=\frac{\mathrm{FSR}}{100}\times \text{Total}.\mathrm{FFM}\times \frac{12.4}{100},$$

where alkali soluble protein of 12.4% total FFM was assumed.^12^, ^13^

Absolute protein breakdown rate (ABR) was estimated as:

$$\mathrm{ABR}\left(g\cdot {\mathrm{day}}^{-1}\right)=\frac{\mathrm{FBR}}{100}\times \text{Total}.\mathrm{FFM}\times \frac{12.4}{100},$$

where fractional breakdown rate (FBR) is calculated as FSR − FGR, with the fractional growth rate (FGR) assumed to be % FFM gain per day over 7 weeks derived by DXA. In addition, the net protein turnover was calculated as ASR − ABR.

#### Muscle RNA, DNA, and protein content

Approximately 15 mg of dry weight muscle was used to determine alkaline soluble protein (ASP), RNA, and DNA content. Initially, 0.2 M PCA was used to homogenize tissue, followed by centrifugation at 11 680 *g*. Pellets were re‐solubilized in 0.3 M NaOH and protein content quantified by spectrophotometry (NanoDrop Lite, Thermo Scientific). Thereafter, the resulting supernatant was used for RNA quantification at 260 nm by spectrophotometry; the pellet was then heated at 70°C for 1 h in 2 M PCA to extract the DNA and centrifuged, and DNA was quantified at 268 nm by spectrophotometry.^12^, ^13^

#### Immunoblotting for anabolic/catabolic signalling

Spectrophotometry was used to determine protein concentrations of sarcoplasmic fractions, and samples were diluted with 3× Laemmli loading buffer (*Table* S2) to 1 mg·mL^−1^, followed by heating at 50°C for 5 min for measuring oxidative phosphorylation (OxPhos) and 95°C for 5 min in order to measure other anabolic and catabolic targets. Precisely 10 μg of samples were loaded onto Criterion XT Bis–Tris–12% SDS‐PAGE gels (Bio‐Rad) for electrophoresis at 185 V for 45 min. After electrophoresis, as previously described,^21^ samples were transferred onto polyvinylidene difluoride (PVDF) membranes for 45 min at 100 V. Subsequently, 2.5% low‐fat milk that was diluted in Tris‐buffered saline Tween‐20 (TBST) was used to soak and block PVDF membranes for 1 h at ambient room temperature and then incubated in the following primary antibodies (*Table* S2) overnight at 4°C (1:2000 dilution in 2.5% BSA in TBS‐T): rabbit, androgen receptor (AR) (#3202), phospho‐protein kinase B (Akt)^Ser473^ (#9271), phospho‐mechanistic target of rapamycin (mTOR)^Ser2448^ (#2971), phospho‐p70 S6 Kinase (p70S6K)^Thr389^ (#9234), phospho‐4E‐BP1^Thr37/46^ (#2855), phospho‐AMP‐activated protein kinase (AMPKα)^Thr172^ (#2531), phospho‐regulatory associated protein of mTOR (Raptor)^Ser792^ (#2083), phospho‐tuberin/TSC2^Thr1462^ (#3617), phospho‐forkhead box O3 (FoxO3a)^Ser253^ (#13129) (all from Cell Signaling Technology, Leiden, The Netherlands), and OxPhos rodent antibody Cocktail (ab110413, Abcam, Cambridge, MA). After overnight incubation, membranes were washed for 3 × 5 min in TBS‐T and soaked in horseradish peroxidase (HRP)‐conjugated secondary antibody [New England Biolabs; 1:2000 in 2.5% bovine serum albumin (BSA) in TBS‐T] for 1 h, before 3 × 5 min washes in TBS‐T. In order to quantify band intensity (Chemidoc MP, Bio‐Rad, Hemel Hempstead, UK), membranes were exposed to Chemiluminescent HRP substrate (Millipore Corp., Billerica, MA, USA) for 5 min. Relative arbitrary units (RAU) were normalized to coomassie‐stained membranes and to cross gel loading control.

#### Gene expression analysis of myogenic, insulin‐like growth factor 1 related, and testosterone processing

Approximately 10 mg of muscle was homogenized, with one stainless steel bead (Tissue Lyser II, Qiagen, UK), for 2 min at frequency of 30 s^−1^ in 500 μL of TRizol (Life Technologies/Thermo Fisher Scientific) to isolate total RNA according to the manufacturer's instructions. A high‐capacity cDNA reverse transcription kit (Life Technologies) was used to reverse‐transcribe 500 ng of total RNA for quantitative reverse transcription PCR. Precisely 1 μL of 1:10 diluted cDNA was added in each well of 384‐optical well plates (Life Technologies). Exon–exon boundary‐specific primers were mixed with SYBR Select Master Mix (LifeTechnologies) and RNase‐free water, and 6 μL of the mixed solution, as well as 1 μL of each cDNA were added to each well, with samples run in triplicate. The ViiATM 7 Real‐Time PCR System (Life Technologies) was used according to the following thermal cycling conditions: 2 min at 50°C; 2 min at 95°C; 40 cycles of 15 s at 95°C; and 60 s at 60°C. The ΔΔCt method was used to quantify target mRNA expression with Peptidylprolylisomerase‐A levels measured to correct for variations in RNA input/cDNA synthesis. Primer sequences for each of the probed genes are listed in *Table* S1.

### Statistical analyses

Data are expressed as mean ± SEM, while normality of distribution was examined using the Kolmogorov–Smirnov test. In addition, two‐way analysis of variance (ANOVA) and repeated measure ANOVA (time) with one between‐subject factor (group) were used to compare the changes during the RET programme both within and between the two (P vs. Z) groups. In addition, independent *t*‐tests were used for comparing fold change between the two groups. Cohen's effect sizes (ESs) were also calculated for significant data. ESs of 0 to <0.20 were considered ‘trivial’, 0.20 to <0.50 were considered ‘small’, 0.50 to <0.80 were ‘medium’, and ≥0.80 were ‘large’. Where significant differences were found using repeated measure ANOVA, a *t*‐test with Bonferroni correction for multiple comparisons was applied. Correlation was assessed using the Pearson's product moment correlation coefficient, and intraclass correlation coefficient (ICC) was used to test reliability of DXA and ultrasound‐related outputs. The significance level was defined as *P* ≤ 0.05, and all of the statistical analyses were performed using GraphPad Prism 7.01 (La Jolla, CA).

## Results

### Baseline characteristics of participants and chemically induced hypogonadism

**Figure 2:**
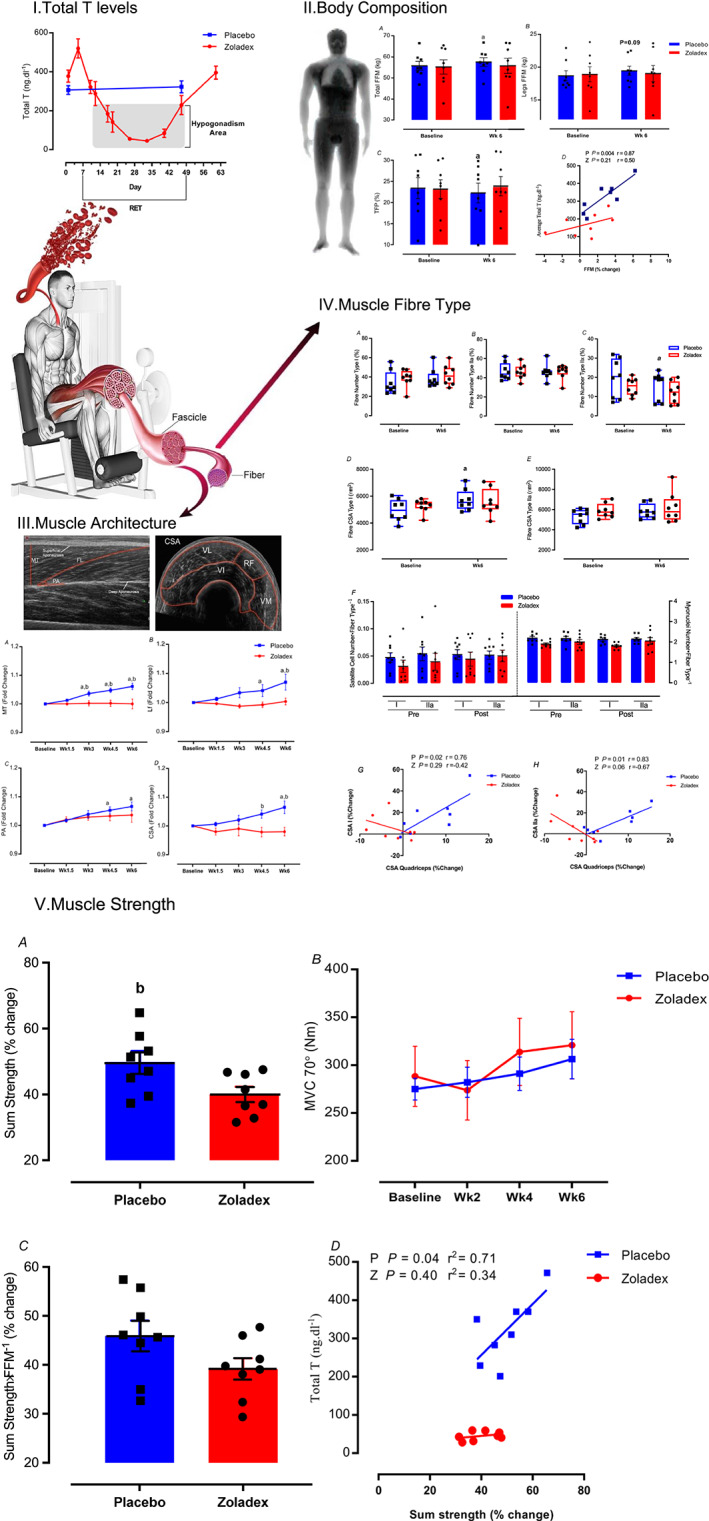
Induced hypogonadism attenuates muscle growth and functional adaptations to RET. Time‐course of changes in (I) plasma T levels in pre vs. post RET in P group and over the study measures in Z group, (II) muscle mass and body composition, (III) muscle architecture, (IV) muscle fibre type features and correlation between fibre type and quadriceps CSA, and (V) changes in dynamic and static strength from baseline to Week 6 in Zoladex (Z) and placebo (P) groups. Values are means ± SEM. a = significantly different from baseline; b = significantly different between the two groups (Z: zoladex, P: placebo), *P* < 0.05. CSA, cross‐sectional area; FFM, fat free mass; Lf, fascicle length; MT, muscle thickness; MVC, maximal voluntary contraction; PA, pennation angle; RET, resistance exercise training; T, testosterone; TFP, total fat percentage. See also *Figures* S1 and S2.

All participants performed activities of daily living and were recreationally active, but had not participated in RET during the previous 12 months. Of those screened and deemed eligible for participation, 16 non‐hypogonadal (morning serum T concentrations of >230 ng·dL^−1^) healthy young men (age: 21.6 ± 0.7 years, weight: 74.3 ± 2.9 kg, height: 1.8 ± 0.1 m; *Table* 1) were assigned in a random double‐blinded fashion to receive either a one‐off: placebo (P, *n* = 8) or GnRH analogue: Goserelin, so called Zoladex (Z) (3.6 mg, *n* = 8) injection before 6 weeks of whole‐body RET. Goserelin prevents the reappearance of luteinizing hormone releasing hormone (LHRH) receptors and, thus, inhibits the secretion of LH from the pituitary gland and consequently testicular production of T.^14^ While most previous human studies^14^ suppressed total T levels to within a castration range, T concentrations in our Z group were intended to be lower than in the P group at all time‐points during RET (*P* < 0.05), while also achieving physiological hypogonadism in order to maintain the translational relevance of the study (i.e. rather than eradicating circulatory T; e.g. Z group: 377 ± 31 ng·dL^−1^ at baseline, 287 ± 61 ng·dL^−1^ at Week 1, and 45 ± 4 ng·dL^−1^ at Week 4 and returning to 229 ± 48 ng·dL^−1^ at the end of RET and finally to the baseline levels after finishing the study) (*Figure* 2I).

### Induced hypogonadism attenuates muscle growth and functional adaptations to resistance exercise training

The DXA (ICC = 0.97) results showed total FFM gains were attenuated significantly following 6 weeks of RET in Z (55.4 ± 2.8 to 55.8 ± 3.1 kg, *P =* 0.61) compared with P (55.9 ± 1.7 to 57.4 ± 1.7 kg, *P =* 0.006, ES = 0.31, treatment‐by‐time interaction: *P* = 0.04, *Figure* 2II.A) with a high correlation between FFM gain and average T concentration over the 6 weeks of RET (*Figure* 2II.D). There was a trend for leg FFM to increase with P only (P: 18.7 ± 0.7 to 19.4 ± 0.7 kg, *P =* 0.09 vs. Z: 18.9 ± 1.1 to 19 ± 1.2 kg, *P* > 0.99, *Figure* 2II.B). Both FFM index (P: 17 ± 0.4 to 17.4 ± 0.5 kg·m^−2^, *P =* 0.007, ES = 0.28 vs. Z:17.5 ± 0.5 to 17.6 ± 0.6 kg·m^−2^, *P =* 0.7, *Figure* S1) and TFP (P: 23.2 ± 2 to 22.1 ± 2%, *P =* 0.04, ES = 0.37 vs. Z: 23 ± 2 to 23.6 ± 2%, *P =* 0.2, treatment‐by‐time interaction: *P* = 0.04, *Figure* 2II.C) adaptation during RET was blunted with chemically induced hypogonadism.

Hypogonadism blunted local muscle remodelling measures (ICC = 0.95) compared with P [muscle thickness (MT), Z: 2.7 ± 0.4 to 2.69 ± 0.36 cm, *P* > 0.99 vs. P: 2.74 ± 0.32 to 2.91 ± 0.32 cm, *P* < 0.0001, ES = 0.48, with a significant difference between two groups (*P* < 0.0001) and treatment‐by‐time interaction effect (*P* < 0.0001); fascicle length (Lf), Z: 7.39 ± 1.1 to 7.42 ± 1.1 cm, *P* > 0.99 vs. P: 7.91 ± 0.87 to 8.44 ± 0.82 cm, *P* < 0.0001, ES = 0.34, with a significant difference between two groups (*P* = 0.003) and treatment‐by‐time interaction effect (*P* = 0.003); pennation angle (PA), Z: 24.4 ± 4.1 to 25.2 ± 4.1°, *P =* 0.32 vs. P: 22.8 ± 1.8 to 24.3 ± 2.27°, *P =* 0.001, ES = 0.36 and CSA, Z: 80.5 ± 15.4 to 78.8 ± 16.4 cm^2^, *P* > 0.99 vs. P: 79.1 ± 11.8 to 83.8 ± 9.1 cm^2^, *P =* 0.005, ES = 0.45, with a significant difference between two groups (*P* = 0.0007) and treatment‐by‐time interaction effect (*P* = 0.007), *Figure* 2III.A–D].

The percentage of fibre type IIx did not change by RET with hypogonadism but decreased in P [Z: 15.1 ± 1.5 to 12.1 ± 2.1%, *P =* 0.15 vs. P: 19.4 ± 3.5 to 15.1 ± 2.5%, *P =* 0.03, ES = 0.48, with a treatment‐by‐time interaction effect (*P* = 0.006)] without changes in type I and IIa in either group (*Figure* 2IV.A–D). Type IIa fibre CSA did not change significantly in any group with RET; but hypogonadism blunted increases in type I fibre CSA (Z: 5276 ± 492 to 5575 ± 965 μm^2^, *P =* 0.47 vs. P: 4953 ± 285 to 5705 ± 275 μm^2^, *P =* 0.01, ES = 0.48, *Figure* 2IV.E,F) with a lack of correlation between quadriceps CSA and type I and IIa CSA with hypogonadism compared with P (*Figure* 2IV.H,I). Finally, there were no significant differences in relative satellite cell number (fibre type I: *P* > 0.99 and fibre type II: *P* > 0.99, *Figure* 2IV.F) and myonuclei number (fibre type I: *P* = 0.67 and fibre type II: *P* > 0.99, *Figure* 2IV.F) between the groups.

Dynamic strength (total 1‐RM across six exercises) was attenuated with hypogonadism compared with P (Z: 40 ± 2.3% vs. P: 49.8 ± 3.3%, *P =* 0.03, ES = 1.4, *Figure* 2V.A) with a lack of correlation between T levels and strength with hypogonadism (*Figure* 2V.D). There were no significant differences in static strength between the groups (MVC at 70°, *P =* 0.22, *Figure* 2V.B), with a similar increase in specific strength (strength per unit area) across the groups (*Figure* 2V.C).

### Hypogonadism attenuates muscle protein turnover increases to resistance exercise training

**Figure 3:**
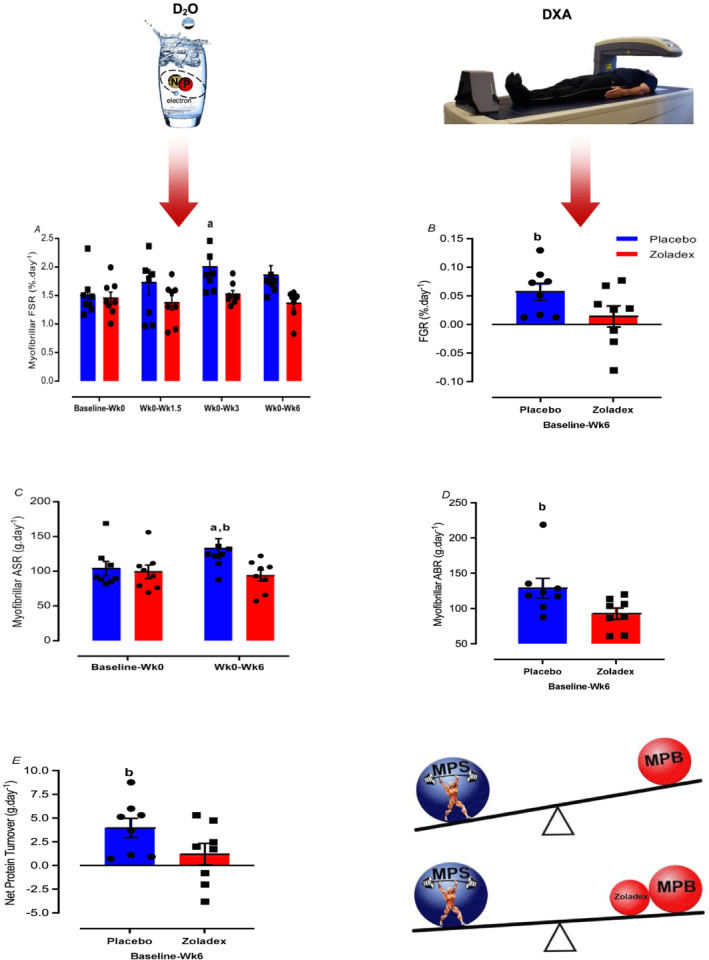
Hypogonadism attenuates muscle protein turnover increases to RET. Values are means ± SEM. a = significantly different from baseline; b = significantly different between the two groups (Z: zoladex, P: placebo), *P* < 0.05. ABR, absolute breakdown rate; ASR, absolute synthetic rate; FGR, fractional growth rate; FSR, fractional synthesis rate; MPS, muscle protein synthesis; RET, resistance exercise training. See also *Figure* S3.

There was a blunted augmentation in cumulative myofibrillar MPS with hypogonadism compared with P over 6 weeks of RET (Z: 1.45 ± 0.11 to 1.5 ± 0.06%·day^−1^, *P =* 0.99 vs. P: 1.5 ± 0.12 to 2 ± 0.15%·day^−1^, *P =* 0.01, ES = 0.97, treatment‐by‐time interaction: *P* = 0.06, *Figure* 3A), in line with findings of blunted FGR in Z compared with P (Z: 0.01 ± 0.01%·day^−1^ vs. P: 0.05 ± 0.01%·day^−1^, *P =* 0.04, ES = 0.43, *Figure* 3B). Furthermore, ASR was not changed a result of RET in Z (Z: 99.9 ± 9.5 to 94.3 ± 7.8 g·day^−1^, *P* > 0.99), but was in P (P: 104.6 ± 10.1 to 133.1 ± 13.9 g·day^−1^, *P =* 0.03, ES = 0.32, *Figure* 3C), with a significant difference between two groups (*P* = 0.03) and treatment‐by‐time interaction effect (*P* = 0.03). In addition, estimated ABR was lower in Z than P during RET (Z: 93.16 ± 7.8 vs. 129.1 ± 13.8 g·day^−1^, *P =* 0.04, ES = 0.92, *Figure* 3D). Finally, net protein turnover with hypogonadism was lower than the P group (3.9 ± 1.1 vs. 1.2 ± 1.1 g·day^−1^, *P =* 0.04, ES = 0.95, *Figure* 3E).

### Hypogonadism impairs the mechanically induced increases in total RNA

There were no changes in ASP or DNA concentrations per dry weight muscle (μg·mg^−1^) in Z or P with RET; nonetheless, total RNA content (Z: *P =* 0.38 vs. P: *P* < 0.0001, ES = 0.75) and RNA:DNA ratio (Z: *P =* 0.31 vs. P: *P =* 0.003, ES = 0.72) increased only in the P group, with significant differences between two groups (*P* < 0.05) and treatment‐by‐time interaction effect (*P* = 0.01). Similarly, RNA:ASP ratio increased in P (*P =* 0.008, ES = 0.63) but not with hypogonadism (*P* > 0.99) over the RET. Finally, the ratio of ASP:DNA, a measure of cell size, did not change in either group (*Table* 2). These results indicate that the increase in translational capacity may be blunted during hypogonadism.

**Table 2:** Mechano‐signals cannot bypass blunted translational *capacity* in hypogonadism after resistance exercise training

	P				Z			
	Baseline	Week 1	Week 3	Week 6	Baseline	Week 1	Week 3	Week 6
ASP content (μg·mg^−1^ dw)	528.1 (21.6)	522.6 (32.9)	564.6 (42)	595.8 (25.4)	592.4 (24.3)	548 (28.7)	581.5 (31.2)	598.2 (24.5)
RNA content (μg·mg^−1^ dw)	3.4 (0.1)	3.8 (0.1)	4.1 (0.1)	5.2 (0.5)^a^ ^,^ ^b^	3.4 (0.1)	3.5 (0.3)	3.5 (0.3)	4.1 (0.4)
DNA content (μg·mg^−1^ dw)	6.8 (0.1)	6.9 (0.3)	7.2 (0.2)	8.1 (0.3)	7.4 (0.4)	7.5 (0.2)	7.4 (0.4)	7.7 (0.7)
RNA:DNA	0.50 (0.01)	0.55 (0.01)	0.57 (0.01)	0.64 (0.04)^a^ ^,^ ^b^	0.47 (0.03)	0.46 (0.04)	0.47 (0.03)	0.53 (0.03)
RNA:ASP	6.5 (0.2)	7.4 (0.4)	7.5 (0.4)	8.9 (1.1)^a^	5.8 (0.4)	6.5 (0.7)	6.1 (0.4)	6.8 (0.5)
ASP:DNA	77.2 (3.2)	75.6 (4.4)	78 (4.6)	74.1 (4.2)	81.1 (4.3)	72.7 (3.1)	78.3 (1.8)	81.2 (8.1)

Values are means (SEM). Total RNA content (Z: *P =* 0.38 vs. P: *P* < 0.0001, ES = 0.75) and RNA:DNA ratio (Z: *P =* 0.31 vs. P: *P =* 0.003, ES = 0.72) increased only in the P group. Similarly, RNA:ASP ratio increased in P (*P =* 0.008, ES = 0.63) but not with hypogonadism (*P* > 0.99) over the RET. dw, dry weight; RET, resistance exercise training.

^a^ Significantly different from baseline.

^b^ Significantly different between two groups, *P* < 0.05.

### Hypogonadism impairs the mechanically induced augmentation in signalling proteins

**Figure 4:**
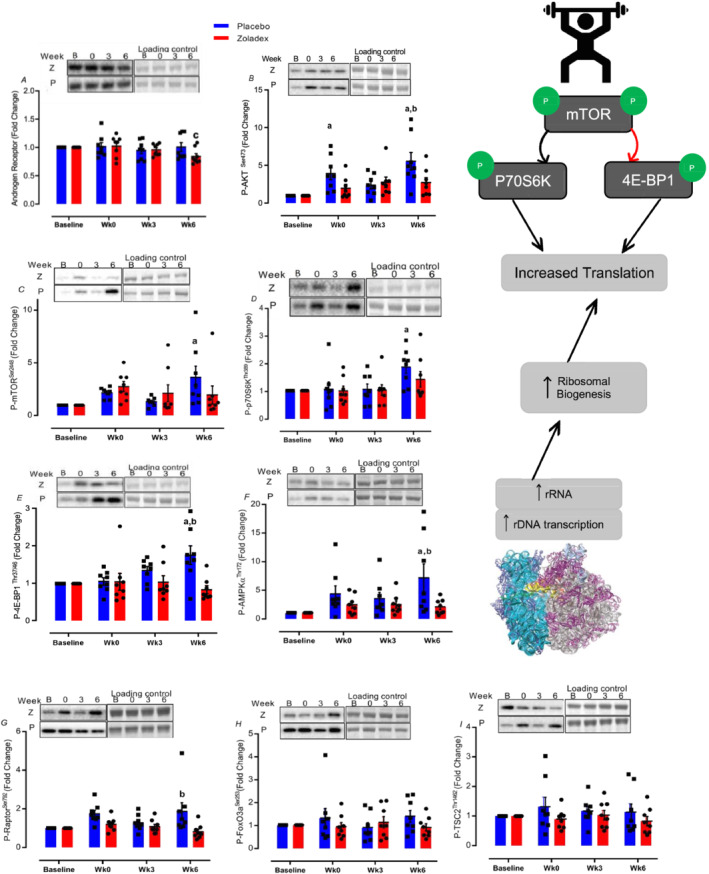
Mechano‐signals cannot bypass blunted translational efficiency in hypogonadism after RET. Values are means ± SEM. a = significantly different from baseline; b = significantly different between the two groups (Z: zoladex, P: placebo), c = significantly different from Week 0, *P* < 0.05. RET, resistance exercise training.

A measure of AR displayed a decrease at 6 weeks in Z, with no significant changes in P group (Z: 0.8 ± 0.01, *P =* 0.01, ES = 0.79 vs. P: 1.01 ± 0.07 fold change, *P* > 0.99, *Figure* 4A). Compared with P, there were blunted increases with hypogonadism in phosphorylation of protein kinase B (AKT^Ser473^) acutely after first RE (Z: 1.99 ± 0.5, *P* > 0.99 vs. P: 3.9 ± 0.8 fold change, *P =* 0.004) and after 6 weeks of RET [Z: 2.74 ± 0.6, *P =* 0.2 vs. P: 5.5 ± 1.1 fold change, *P* < 0.001, ES = 0.54, with significant differences between two groups (*P =* 0.01) and treatment‐by‐time interaction: *P =* 0.02, *Figure* 4B], and mammalian target of rapamycin (mTOR^Ser2448^) [Z: 1.9 ± 0.8, *P* > 0.99 vs. P: 3.6 ± 1 fold change, *P =* 0.002, ES = 0.53, with a trend in treatment‐by‐time interaction effect (*P =* 0.06), *Figure* 4C], and p70S6K^Thr389^ (Z: 1.4 ± 0.2, *P =* 0.4 vs. P: 1.8 ± 0.2 fold change, *P =* 0.003, ES = 0.8, *Figure* 4D) and inhibition of 4EBP1^Thr37/46^ [Z: 0.8 ± 0.1, *P* > 0.99 vs. P: 1.7 ± 0.2 fold change, *P =* 0.002, ES = 0.75, with significant differences between two groups (*P =* 0.002) and treatment‐by‐time interaction: *P =* 0.007, *Figure* 4E] acutely after RE. Compared with P, activation of adenosine monophosphate‐activated protein kinase (AMPKα^Thr172^) (Z: 2.1 ± 0.4, *P* > 0.99 vs. P: 7.1 ± 2.4 fold change, *P =* 0.001, ES = 0.68, *Figure* 4F) and regulatory‐associated protein of mTOR (Raptor^Ser792^) (Z: 0.8 ± 0.1, *P* > 0.99 vs. P:1.8 ± 0.4 fold change, *P =* 0.01, ES = 0.59, *Figure* 4G) was attenuated with hypogonadism acutely after 6 weeks of RET. There were no significant changes in phosphorylation of forkhead box O3 (FoxO3a)^Ser253^ and tuberin/TSC2^Thr1462^ at 6 weeks in either group (*P* > 0.05, *Figure* 4H,I). Further, there was no association between hypogonadism and cellular bioenergetics during RET at translational levels (*Figure* S4).

### Hypogonadism impaired resistance exercise training‐induced myogenic and androgenic, but not mitochondrial adaptations

**Figure 5:**
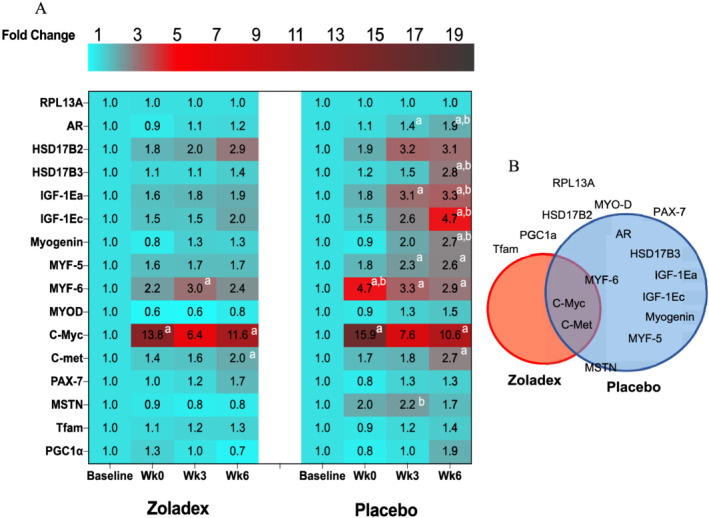
Hypogonadism impaired RET‐induced myogenic and androgenic, but not mitochondrial adaptations. *(A)* Heatmap of the changes in T metabolism, anabolism, myogenic, and myogenesis inhibitor gene expression from baseline to Week 6 of RET in Z and P. *(B)* Venn diagram of the overlap of differentially expressed genes briefly increased in Z, P, both, or neither. Values are means of fold changes normalized to a housekeeping gene (i.e. RPL13A), which exhibited high transcriptional stability over the study (between groups: *P* = 0.8; within both groups: *P* > 0.9). a = significantly different from baseline; b = significantly different between the two groups (Z: zoladex, P: placebo), *P* < 0.05. RET, resistance exercise training.

Compared with P, *AR* (Z: *P* > 0.99 vs. P: *P* < 0.0001, ES = 0.85, treatment‐by‐time interaction: *P =* 0.006), *HSD17β3* (Z: *P* > 0.99 vs. P: *P =* 0.007, ES = 1.04, treatment‐by‐time interaction: *P =* 0.02) was blunted after 6 weeks of RET with hypogonadism, with significant differences between two groups (*P* < 0.05). In addition, *IGF‐1Ea* (Z: *P =* 0.5 vs. P: *P =* 0.0005, ES = 0.72, treatment‐by‐time interaction: *P =* 0.04) and *IGF‐1Ec* (Z: *P* > 0.99 vs. P: *P =* 0.0005, ES = 0.68, treatment‐by‐time interaction: *P =* 0.08) expressions increased in P but were blunted with hypogonadism after 6 weeks of RET, with significant differences between two groups (*P* < 0.05). While mRNA expression of select myogenesis‐related genes, that is, *myogenin* (Z: *P* > 0.99 vs. P: *P =* 0.002, ES = 0.72, treatment‐by‐time interaction: *P =* 0.05), *Myf‐5* (Z: *P =* 0.34 vs. P: *P =* 0.0001, ES = 0.81, treatment‐by‐time interaction: *P =* 0.07), and *Myf‐6* (Z: *P =* 0.11 vs. P: *P =* 0.01, ES = 0.79, treatment‐by‐time interaction: *P =* 0.01), were not changed with hypogonadism compared with P, *C‐met* (Z: *P =* 0.03 vs P: *P* < 0.0001, ES = Z:0.77, P:0.82) and *C‐Myc* (Z: *P =* 0.007 vs. P: *P =* 0.01, ES = Z:0.74, P:0.74) increased in both groups, with no significant changes in *MYOD* and *PAX‐7* (*P* > 0.05). Nevertheless, a measure of myostatin (*MSTN*) displayed a trend to increase by 3 weeks in P (*P =* 0.01, ES = 0.55), with no significant changes at 6 weeks in either group (*P* > 0.05, *Figure* 5). Finally, there were no differences in the gene expression of mitochondrial transcription factor A (*Tfam*) or peroxisome proliferator‐activated receptor γ co‐activator‐1α (*PGC‐1α*) in either group (*P* > 0.05).

## Discussion

In the present study, chemically induced hypogonadism blunted augmentation in whole‐body FFM gains after 6 weeks of RET, a well‐established model of hypertrophy,^18^ with DXA data showing the impact of hypogonadism on different body composition components [i.e. FFM and total fat percentage (TFP)] (*Figure* 2II). The role of T in regulating FFM gains with RET has been contentious. For instance, West and Phillips reported that 12 weeks of RET increased FFM without altering endogenous T levels, concluding that changes in endogenous T levels did not impact RET‐induced muscular adaptations.^16^ Conversely, 10 weeks of endogenous T suppression decreased FFM gains in young men,^22^ while in another study, T depletion to castration range for 12 weeks blunted muscle hypertrophy during 8 weeks of RET in young men.^14^ We conclude that induction of hypogonadism blunts whole‐body muscle hypertrophy during RET. We also sought to determine changes in muscle architecture, focusing on the muscle undergoing sampling biopsies to seek molecular explanations for our findings. Reflecting whole‐body data, RET‐induced gains in MT, Lf and PA of the *vastus lateralis* (VL), and CSA of the quadriceps were blunted in the Z group. Increases in MT is an expected adaptive response to RET and it can be attributed to increases in both PA and Lf.^23^ However, the fact that no changes in PA or Lf after RET were reported in older men with lower T levels after 6^12^, ^13^ or 16 weeks of RET,^24^ and the association between low T levels and decline in MT,^25^ may underline T's key role in RET‐induced remodelling of thigh muscles. Indeed, our state of induced hypogonadism attenuated these aspects of muscle architectural adaptation and thigh muscle hypertrophic remodelling.

We also sought to investigate muscle fibre level remodelling and that noted hypertrophy (CSA) of type I fibres and a decrease in the relative ratio of type IIx fibres were blunted with hypogonadism. There was also a link between fibre type CSA, muscle hypertrophy, and T levels. Previous studies in young men have shown that fibre CSA (type I and II) increased after 12 weeks of RET and that this global hypertrophic response in both slow and fast muscle fibres has been attributed to T levels.^26^ Furthermore, injections of T (25–600 mg·week^−1^) for 20 weeks have been associated with increases in type I and II fibre CSA in young men.^27^ Type II fibres are more sensitive to physiological alteration; for example, loss of muscle mass with ageing is primarily due to the specific atrophy of muscle fibre type II, which is associated with lower satellite cell and myonuclei number.^28^ Indeed, satellite cell proliferation and the subsequent incorporation of their differentiated progeny (i.e. newly formed myonuclei) are essential to allow muscle fibre type II hypertrophy.^29^ However, we did not observe any tendency towards changes in type IIa CSA with RET or hypogonadism, in line with lack of changes in satellite cell and myonuclei numbers. It seems, in our younger participants, the maximum amount of cytoplasm that can be controlled by one myonucleus, referred to as the ‘myonuclear domain’ ceiling,^30^ was already reached in type II fibres.^28^, ^29^, ^31^ In support of this proposition, fibre type CSA and proportion was unchanged after 30 and 90 days of RET in both young^31^ and older men.^32^ Conversely, fibre type II CSA increased while fibre type I CSA decreased after 12 weeks of RET,^33^ representing a significant discrepancy in the literature about the impacts of RET on myofibre adaptations.^29^, ^32^ However, our data suggest that levels of endogenous T, in healthy young men, can be considered to be of paramount importance for the muscular adaptation (both global and local remodelling) to RET.

Muscular performance is the culmination of neural and muscle structural elements and is the most important attribute to physical function, which increased after 8 weeks of RET in young men.^34^ Herein, we established that hypogonadism attenuated gains in total dynamic (1‐RM) strength, which were strongly correlated with endogenous T levels (*Figure* 2V.D), as was shown after 12 weeks of RET in middle‐aged men.^35^ It had been reported that 12 weeks of chemical T castration coupled with 8 weeks of RET blunted strength gains in young men,^14^ through attenuating FFM gains rather than neural remodelling (*Figure* 2V.C). However, in contrast, other previous studies have shown that there may be a neural component to the effects of T, for example, increasing neurotransmitter synthesis, leading to increased strength and higher threshold motor unit discharge and less force fluctuation, which results in the recruitment of larger/faster motor units (fast twitch fibres).^36^ Our data illustrate that muscle mass gains were correlated with endogenous T levels; however, the lack of difference between the two experimental groups in specific strength per unit gains suggest blunted RET‐induced FFM gains with hypogonadism were likely the major effector of performance deficits rather than other factors, for example, neural adaptations.

In comparison with the P group, hypogonadism attenuated MPS during RET while concomitantly blunting FBRs; crucially, net balance calculations illustrated that MPS > MPB in both groups (*Figure* 3). MPB is an important contributor to muscle remodelling and protein turnover, increasing after bouts of RE but to a lesser extent than MPS.^3^ The logical extrapolation of a sustained increase in net muscle protein deposition after RET is an increase in FFM and strength,^12^, ^13^, ^37^ in line with the gains in mass and strength in the P group in our study. Links between T and MPS have been long recognized. For instance, castration induced T withdrawal suppressed MPS components, decreasing muscle mass and strength in mice.^38^, ^39^ Similarly, castration coupled to ‘rescue’ T replacement therapy for 2 weeks in mice indicated T as a key regulator of muscle protein turnover.^38^ However, in humans, it has previously been suggested that T supplementation may enhance net protein balance via an increase in MPS, with a trend towards an increase in MPB in young men.^40^ Conversely, 10 weeks of T suppression to within a hypogonadal range decreased whole‐body protein synthesis and breakdown and IGF‐1 expression, leading to decreased muscle strength and mass in young men^22^; therefore, it may be expected that T removal may result in a decrease in MPB in humans. Herein, physiological endogenous T levels coupled to RET increased net protein accretion accompanied by increases in both MPS and MPB, with MPS > MPB indicating the central role of endogenous T in regulating RET‐induced muscle protein turnover.^37^ These findings highlight that T play a permissive role for RET‐induced increases in muscle protein turnover. To seek the underlying molecular transducers regulating this attenuated adaptive response, we investigated factors controlling intracellular protein turnover (i.e. protein translation, available mRNA, and transcriptional capacity), which reportedly have major effects on the rate of muscle hypertrophy.^13^

Given that ribosomal RNA constitutes the major portion of total RNA (i.e. ~85%), an increase in total RNA content indicates reduced ribosomal (r) degradation (ribophagy)^41^ and/or enhanced r biogenesis and translational capacity through activation of rDNA transcription.^42^ rDNA transcription, which is the major rate limiting factor for ribosome production, is deemed essential for myofibre growth^42^ and also in hypertrophic responses to RE. In support of this is the observation of *acute* RE‐induced increases in ribosomal RNA content increasing the ‘capacity’ for protein synthesis and promoting net protein balance, which are critical for anabolic potential and hypertrophy during RET.^12^, ^13^ Indeed, ribosomal biogenesis is likely an important component of RET‐induced muscle hypertrophy during longer‐term RET in humans, that is, rRNA and ribosomal biogenesis increase after 8 weeks of RET in young men,^43^ while rRNA was claimed to be a key indicator of ribosomal biogenesis and a molecular transducer of muscle growth over 12 weeks of RET in young adults.^44^ Here, we demonstrate that increases in RNA content and RNA:DNA ratio, a reflection of ribosomal capacity (quantity of ribosome) for protein synthesis, were blunted in the Z group after RET. Similarly, the RNA:ASP (ASP: alkaline soluble protein) ratio, a measure of ribosomal efficiency,^12^ was attenuated with hypogonadism (*Table* 2). We previously showed that those who had lower normal T levels exhibited blunted ribosomal biogenesis and capacity as molecular transducers of 6 weeks of whole‐body and unilateral RET,^12^, ^13^ and also, total muscle RNA was decreased in orchiectomized rats.^39^ Thus, increases in total RNA content per ‘cellular unit’ (RNA:DNA ratio), an indicator of ribosomal abundance, and total RNA:ASP ratio, an index of synthetic capacity,^1^ require eugonadal levels of T, demonstrating the inability of RET to augment translational activity in a hypogonadal milieu. To conclude, chemically induced hypogonadism blunted translational capacity, which may be partly responsible for the ‘anabolic resistance’ of muscle to RET with Z.

We next investigated aspects regulating the translational ‘efficiency’ of ribosomes corresponding to MPS, namely, the phosphorylation of key (e.g. mTOR) adaptive signalling networks at ‘snap‐shot’ periods during the study (i.e. first and last RE bouts) in both placebo and Zoladex groups. In doing so, we are quantifying the effects of *acute* RE on the background of *chronic* eugonadal and hypogonadal states. As such, a notable interpretative limitation is that we are unable to determine if measured effects are due to *acute* effects of RE, and/or the *chronic* background of our interventions. Nonetheless, in making these measures, we noted ‘RE‐induced’ activation of numerous molecular transducers of muscle growth were blunted in hypogonadism, and in a similar fashion to individuals who have lower endogenous levels (i.e. older vs. young men, although this is speculative)^12^, ^45^—as well as in castrated mice.^38^ The anabolic effects of androgens on skeletal^46^ and cardiac^47^ muscle are reported to be mediated via mTORC1 pathways, and it is speculated that the generalized impaired activation of, for example, mTORC1, ERK1/2, and MAPK growth pathways^45^ could have resulted in blunted muscle fibre hypertrophy and RE‐induced increases in total RNA content (*Table* 2) observed. Finally, some studies report that castration is associated with an increase in MPB pathways in mice (e.g. AMPKα^38^). However, we show that RET activates both anabolic and catabolic pathways with MPS > MPB and with hypogonadism blunting both MPS (e.g. mTOR pathway) and MPB, which lead to reduce the absolute positive shift in protein balance after RE bouts^48^ and attenuating increases in muscle mass over RET.

We show steroidogenesis enzyme expression to be blunted after 6 weeks of RET in a hypogonadal environment. Single bout (i.e. acute) of RE did not change muscle steroidogenic enzyme expression in young men.^49^ Although 12 weeks of RET increased intramuscular steroidogenic enzyme levels in young men^50^ and also restored age‐associated reductions in muscle sex steroid hormone levels and muscle steroidogenic enzyme expression in older men,^49^ which correlated with increases in muscle strength and CSA,^49^ we have already shown that 6 weeks of RET was not a potent enough stimuli to overcome age‐associated andropause in older men.^12^, ^13^ Herein, hypogonadism attenuated the RET induction of *AR* mRNA and protein levels possibly resulting in lower muscle T processing capacity due to lower T in the circulation.^51^ Because androgen‐bound AR alters mRNA expression of many molecular exercise transducers, for example, myogenesis,^51^ and is associated with triggering anabolic kinase signalling, that is, Akt, the fact that its expression is blunted at both the gene and protein levels with hypogonadism would suggest translational and transcriptional links between endogenous T and changes in MPS, FFM, and strength in this present study and others.^12^, ^49^ We also demonstrate that anabolic and myogenic‐related gene expression are blunted during chemically induced hypogonadism even during RET, that is, represented by blunted *IGF‐1Ec* and *IGF‐1Ea* expression, because *IGF‐1Ea* is purported to correlate with increased translation and promote myogenesis differentiation,^52^ while *IGF‐1Ec* expression is associated with transcriptional activity and also initiation of satellite cell proliferation.^52^ T suppression for 10 weeks decreased *IGF‐1* mRNA concentration in young men,^22^ indicating a possible link between the IGF‐1 axis and T levels, and other anabolic genes.^51^ In the present study, in line with past work in older adults,^13^, ^52^ we also show that hypogonadism is associated with the attenuation of the expression of myogenic regulatory factors (MRFs) after 6 weeks of RET, in line with blunting of both MPS and whole‐body hypertrophy. This supports previous work demonstrating a pro‐myogenic role for T; for example, T increased the transformation of pluripotent precursor cells towards a myogenic lineage.^39^ We report an augmentation in *C‐Myc* expression in both experimental groups. It is reported *C‐Myc* expression is highly sensitive to RET^13^ and ablation of T decreased *C‐Myc* mRNA in orchiectomized rats^39^; however, hypogonadal T levels in our study do not blunt its increased expression during RET. This seems an unlikely feature of blunted muscle hypertrophy in our study, while we showed chemically induced hypogonadism blunted transcriptional capacity components during 6 weeks of RET. However, it is reported that muscle growth is characterized more by translational, than transcriptional regulation.^2^

Finally, because T up‐regulates cellular bioenergetics via increases in ATP synthesis and mitochondrial content that are associated with increases in mitochondrial biogenesis and androgen deficiency contributes to the pathophysiology of ‘mitochondrial dysfunction’,^11^ we investigated any potential impacts of hypogonadism during RET upon mitochondrial adaptations (i.e. mitochondrial biogenesis and function). It has been reported that RET stimulates mitochondrial biogenesis; that is, 10 weeks of RET augmented mitochondrial biogenesis, albeit to a lesser extent than endurance training.^53^ In our study, neither RET nor hypogonadism were associated with alteration in cellular bioenergetics, as determined by mitochondrial oxidative phosphorylation (OxPhos) blots (*Figure* S4). In line with this, we observed no differences in the gene expression of mitochondrial transcription factor A (*Tfam*) or peroxisome proliferator‐activated receptor γ co‐activator‐1α (*PGC‐1α*). Thus, while castration‐induced AR changes were associated with decreases in *Tfam and PGC‐1α* at the level of both protein and gene expression in animal models,^11^ we observed no association between hypogonadism and cellular bioenergetics during RET at either the transcriptional (*Figure* 5) or translational levels (*Figure* S4). As such, impaired cellular bioenergetics and mitochondrial biogenesis induction by hypogonadism would both seem unlikely causes of the blunted muscle hypertrophic response we observe in this study.

We acknowledge limitations to our study. Firstly, not measuring corresponding total protein pools alongside phosphorylated signalling proteins is considered as limitation of our study though does not detract from our data showing the importance of T in bolstering MPS‐related signalling activity and by extension, exercise‐induced muscle growth during RET. Also, biopsy timings within this study reflect acute responses to RE and how these adapt over time and do not demonstrate how resting levels of gene and signalling protein activation may change with RET. Thus, further time‐course biopsies (e.g. at resting stage over RET) would lead to a more complete understanding of the molecular responses to RET adjuvant to hypogonadism.

## Conclusions

T suppression via a single GnRh analogue injection was well tolerated by the volunteers and demonstrated the negative impact of hypogonadism on muscle hypertrophic programming via blunting the normal responses of muscle molecular transducers, that is, anabolic signalling proteins (mTOR pathway), T processing enzymes (HSDs), pro‐myogenic gene expression, which result in an increase in the capacity for protein synthesis (e.g. RNA biogenesis), and consequently MPS. Thereby, we demonstrate the importance of endogenous T in adopting a permissive role in regulating both muscle protein turnover and hypertrophy, which is not rescuable via mechano‐transduction alone. From a gender perspective, T is also important in regulating muscle mass and strength in women, with acute increases in T levels in women being reported in some studies in response to RE but not all.^54^ However, further studies are needed to investigate the effects of endogenous T upon muscle quality/programming in premenopausal women.

## Funding

This work was supported by the Medical Research Council (grant numbers MR/R502364/1 and MR/P021220/1) as part of the MRC‐Versus Arthritis Centre for Musculoskeletal Ageing Research awarded to the Universities of Nottingham and Birmingham, and the National Institute for Health Research, Nottingham Biomedical Research Centre.

## Conflict of interest

The authors have declared that no conflict of interest exists.

## Supporting information


**Figure S1** (related to Figure 2). Induced hypogonadism attenuates muscle growth and functional adaptations to RET. Values are means ± SEM. **a** = significantly different from baseline; **b** = significantly different between the two groups (Z: zoladex, P: placebo), *P* < 0.05. FFM: fat free mass, TFM: total fat mass, CSA: cross sectional area, MVC: maximal voluntary contraction, RET: resistance exercise training.
**Figure S2 (related to Figure 2).** Muscle cross‐section stained for fibre type‐specific identification of satellite cells. Satellite cells are stained brown with Pax7 antibody, whereas laminin and type I fibres are stained with fluorescent green and type IIA fibres with fluorescent red. Myonuclei are stained blue (DAPI).
**Figure S3 (related to Figure 3).** Hypogonadism attenuates muscle protein turnover increases in response to RET. Values are means ± SEM. **b** = significantly different between two groups (Z: zoladex, P: placebo), *P* < 0.05. FBR: fractional breakdown rate, RET: resistance exercise training.
**Figure S4 (related to Figure 4).** Mechano‐signals cannot bypass blunted translational efficiency in hypogonadism after RET. Values are means ± SEM. **a** = significantly different from baseline; **b** = significantly different between the two groups (Z: zoladex, P: placebo), *P* < 0.05. NDUFB8: NADH dehydrogenase [ubiquinone] 1 beta subcomplex subunit 8, SDHB: Succinate dehydrogenase [ubiquinone] iron–sulfur subunit, UQCRC2: Cytochrome b‐c1 complex subunit 2, MTCO1: Mitochondrially encoded cytochrome c oxidase I, ATP5A: ATP synthase F1 subunit alpha, RET: resistance exercise training.
**Table S1.** Primer sequences for each of the probed genes used in PCR.
**Table S2.** Key resources.

## References

[1] McCarthy JJ , Esser KA . Anabolic and catabolic pathways regulating skeletal muscle mass. Curr Opin Clin Nutr Metab Care 2010;13:230–235. 20154608 10.1097/MCO.0b013e32833781b5 PMC2877703

[2] Pereira MG , Dyar KA , Nogara L , Solagna F , Marabita M , Baraldo M , et al. Comparative analysis of muscle hypertrophy models reveals divergent gene transcription profiles and points to translational regulation of muscle growth through increased mTOR signaling. Front Physiol 2017;8:968. 29255421 10.3389/fphys.2017.00968 PMC5723052

[3] Tipton KD , Hamilton DL , Gallagher IJ . Assessing the role of muscle protein breakdown in response to nutrition and exercise in humans. Sports Med (Auckland, NZ) 2018;48:53–64. 10.1007/s40279-017-0845-5 PMC5790854 29368185

[4] Léger B , Cartoni R , Praz M , Lamon S , Dériaz O , Crettenand A , et al. Akt signalling through GSK‐3β, mTOR and Foxo1 is involved in human skeletal muscle hypertrophy and atrophy. J Physiol 2006;576:923–933. 16916907 10.1113/jphysiol.2006.116715 PMC1890416

[5] Thoreen CC , Chantranupong L , Keys HR , Wang T , Gray NS , Sabatini DM . A unifying model for mTORC1‐mediated regulation of mRNA translation. Nature 2012;485:109–113. 22552098 10.1038/nature11083 PMC3347774

[6] Bond P . Regulation of mTORC1 by growth factors, energy status, amino acids and mechanical stimuli at a glance. J Int Soc Sports Nutr 2016;13:1–11. 26937223 10.1186/s12970-016-0118-y PMC4774173

[7] Miyazaki M , McCarthy JJ , Fedele MJ , Esser KA . Early activation of mTORC1 signalling in response to mechanical overload is independent of phosphoinositide 3‐kinase/Akt signalling. J Physiol 2011;589:1831–1846. 21300751 10.1113/jphysiol.2011.205658 PMC3099033

[8] Deldicque L , Atherton P , Patel R , Theisen D , Nielens H , Rennie MJ , et al. Decrease in Akt/PKB signalling in human skeletal muscle by resistance exercise. Eur J Appl Physiol 2008;104:57–65. 18535836 10.1007/s00421-008-0786-7

[9] Schoenfeld BJ . The mechanisms of muscle hypertrophy and their application to resistance training. J Strength Cond Res 2010;24:2857–2872. 20847704 10.1519/JSC.0b013e3181e840f3

[10] Kraemer WJ , Ratamess NA , Hymer WC , Nindl BC , Fragala MS . Growth hormone(s), testosterone, insulin‐like growth factors, and cortisol: roles and integration for cellular development and growth with exercise. Front Endocrinol 2020;11:1–25. 10.3389/fendo.2020.00033 PMC7052063 32158429

[11] Traish AM , Abdallah B , Yu G . Androgen deficiency and mitochondrial dysfunction: implications for fatigue, muscle dysfunction, insulin resistance, diabetes, and cardiovascular disease. Horm Mol Biol Clin Invest 2011;8:431–444. 10.1515/HMBCI.2011.132 25961343

[12] Brook MS , Wilkinson DJ , Mitchell WK , Lund JN , Phillips BE , Szewczyk NJ , et al. Synchronous deficits in cumulative muscle protein synthesis and ribosomal biogenesis underlie age‐related anabolic resistance to exercise in humans. J Physiol 2016;594:7399–7417. 27654940 10.1113/JP272857 PMC5157077

[13] Gharahdaghi N , Rudrappa S , Brook MS , Idris I , Crossland H , Hamrock C , et al. Testosterone therapy induces molecular programming augmenting physiological adaptations to resistance exercise in older men. J Cachexia Sarcopenia Muscle 2019;10:1276–1294. 31568675 10.1002/jcsm.12472 PMC6903447

[14] Kvorning T , Andersen M , Brixen K , Madsen K . Suppression of endogenous testosterone production attenuates the response to strength training: a randomized, placebo‐controlled, and blinded intervention study. Am J Physiol Endocrinol Metab 2006;291:1325–1332. 10.1152/ajpendo.00143.2006 16868226

[15] West DW , Burd NA , Tang JE , Moore DR , Staples AW , Holwerda AM , et al. Elevations in ostensibly anabolic hormones with resistance exercise enhance neither training‐induced muscle hypertrophy nor strength of the elbow flexors. J Appl Physiol 2009;108:60–67. 19910330 10.1152/japplphysiol.01147.2009 PMC2885075

[16] West DW , Phillips SM . Associations of exercise‐induced hormone profiles and gains in strength and hypertrophy in a large cohort after weight training. Eur J Appl Physiol 2012;112:2693–2702. 22105707 10.1007/s00421-011-2246-z PMC3371329

[17] West DW , Kujbida GW , Moore DR , Atherton P , Burd NA , Padzik JP , et al. Resistance exercise‐induced increases in putative anabolic hormones do not enhance muscle protein synthesis or intracellular signalling in young men. J Physiol 2009;587:5239–5247. 19736298 10.1113/jphysiol.2009.177220 PMC2790261

[18] DeFreitas JM , Beck TW , Stock MS , Dillon MA , Kasishke PR . An examination of the time course of training‐induced skeletal muscle hypertrophy. Eur J Appl Physiol 2011;111:2785–2790. 21409401 10.1007/s00421-011-1905-4

[19] Kadi F , Hägg G , Håkansson R , Holmner S , Butler‐Browne GS , Thornell L‐E . Structural changes in male trapezius muscle with work‐related myalgia. Acta Neuropathol 1998;95:352–360. 9560012 10.1007/s004010050810

[20] Wilkinson DJ , Franchi MV , Brook MS , Narici MV , Williams JP , Mitchell WK , et al. A validation of the application of D_2_O stable isotope tracer techniques for monitoring day‐to‐day changes in muscle protein subfraction synthesis in humans. Am J Physiol Endocrinol Metab 2014;306:E571–E579. 24381002 10.1152/ajpendo.00650.2013 PMC3948971

[21] Bass JJ , Wilkinson DJ , Rankin D , Phillips BE , Szewczyk NJ , Smith K , et al. An overview of technical considerations for Western blotting applications to physiological research. Scand J Med Sci Sports 2017;27:4–25. 27263489 10.1111/sms.12702 PMC5138151

[22] Mauras N , Hayes V , Welch S , Rini A , Helgeson K , Dokler M , et al. Testosterone deficiency in young men: marked alterations in whole body protein kinetics, strength, and adiposity. J Clin Endocrinol Metabol 1998;83:1886–1892. 10.1210/jcem.83.6.4892 9626114

[23] Blazevich AJ , Sharp NC . Understanding muscle architectural adaptation: macro‐and micro‐level research. Cells Tissues Organs 2005;181:1–10. 16439814 10.1159/000089964

[24] Selva Raj I , Bird S , Westfold B , Shield A . Effects of eccentrically biased versus conventional weight training in older adults. Med Sci Sports Exerc 2012;44:1167–1176. 22143107 10.1249/MSS.0b013e3182442ecd

[25] Fujiwara K , Asai H , Toyama H , Kunita K , Yaguchi C , Kiyota N , et al. Changes in muscle thickness of gastrocnemius and soleus associated with age and sex. Aging Clin Exp Res 2010;22:24–30. 19920407 10.1007/BF03324811

[26] Kadi F , Eriksson A , Holmner S , Thornell LE . Effects of anabolic steroids on the muscle cells of strength‐trained athletes. Med Sci Sports Exerc 1999;31:1528–1534. 10589853 10.1097/00005768-199911000-00006

[27] Sinha‐Hikim I , Artaza J , Woodhouse L , Gonzalez‐Cadavid N , Singh AB , Lee MI , et al. Testosterone‐induced increase in muscle size in healthy young men is associated with muscle fiber hypertrophy. Am J Physiol Endocrinol Metab 2002;283:154–164. 10.1152/ajpendo.00502.2001 12067856

[28] Verdijk LB , Gleeson BG , Jonkers RA , Meijer K , Savelberg HH , Dendale P , et al. Skeletal muscle hypertrophy following resistance training is accompanied by a fiber type–specific increase in satellite cell content in elderly men. J Gerontol A Biomed Sci Med Sci 2009;64:332–339. 10.1093/gerona/gln050 PMC2655000 19196907

[29] Petrella JK , J‐s K , Cross JM , Kosek DJ , Bamman MM . Efficacy of myonuclear addition may explain differential myofiber growth among resistance‐trained young and older men and women. Am J Physiol Endocrinol Metab 2006;291:937–946. 10.1152/ajpendo.00190.2006 16772322

[30] Kadi F . Adaptation of human skeletal muscle to training and anabolic steroids. Acta Physiol Scand 2000;168:4–53. 10717767

[31] Kadi F , Schjerling P , Andersen LL , Charifi N , Madsen JL , Christensen LR , et al. The effects of heavy resistance training and detraining on satellite cells in human skeletal muscles. J Physiol 2004;558:1005–1012. 15218062 10.1113/jphysiol.2004.065904 PMC1665027

[32] Mackey A , Esmarck B , Kadi F , Koskinen S , Kongsgaard M , Sylvestersen A , et al. Enhanced satellite cell proliferation with resistance training in elderly men and women. Scand J Med Sci Sports 2007;17:34–42. 17305939 10.1111/j.1600-0838.2006.00534.x

[33] Kryger A , Andersen J . Resistance training in the oldest old: consequences for muscle strength, fiber types, fiber size, and MHC isoforms. Scand J Med Sci Sports 2007;17:422–430. 17490465 10.1111/j.1600-0838.2006.00575.x

[34] Tsitsilonis S , Panayiotis CE , Athanasios MS , Stavros KK , Ioannis VS , George A , et al. Anabolic androgenic steroids reverse the beneficial effect of exercise on tendon biomechanics: an experimental study. Foot Ankle Surg 2014;20:94–99. 24796826 10.1016/j.fas.2013.12.001

[35] Häkkinen K , Pakarinen A . Serum hormones and strength development during strength training in middle‐aged and elderly males and females. Acta Physiol Scand 1994;150:211–219. 8191900 10.1111/j.1748-1716.1994.tb09678.x

[36] Bellew J . The effect of strength training on control of force in older men and women. Aging Clin Exp Res 2002;14:35–41. 12027150 10.1007/BF03324415

[37] Gharahdaghi N , Phillips BE , Szewczyk NJ , Smith K , Wilkinson DJ , Atherton PJ . Links between testosterone, oestrogen, and the growth hormone/insulin‐like growth factor axis and resistance exercise muscle adaptations. Front Physiol 2021;11:1814. 10.3389/fphys.2020.621226 PMC7844366 33519525

[38] Serra C , Sandor NL , Jang H , Lee D , Toraldo G , Guarneri T , et al. The effects of testosterone deprivation and supplementation on proteasomal and autophagy activity in the skeletal muscle of the male mouse: differential effects on high‐androgen responder and low‐androgen responder muscle groups. Endocrinology 2013;154:4594–4606. 24105483 10.1210/en.2013-1004 PMC3836062

[39] Mobley C , Mumford P , Kephart W , Conover C , Beggs L , Balaez A , et al. Effects of testosterone treatment on markers of skeletal muscle ribosome biogenesis. Andrologia 2016;48:1055–1065. 10.1111/and.12539 26781353

[40] Ferrando AA , Tipton KD , Doyle D , Phillips SM , Cortiella J , Wolfe RR . Testosterone injection stimulates net protein synthesis but not tissue amino acid transport. Am J Physiol Endocrinol Metab 1998;275:E864–E871. 10.1152/ajpendo.1998.275.5.E864 9815007

[41] Figueiredo VC , D'Souza RF , Van Pelt DW , Lawrence MM , Zeng N , Markworth JF , et al. Ribosome biogenesis and degradation regulate translational capacity during muscle disuse and reloading. J Cachexia Sarcopenia Muscle 2021;12:130–143. 33231914 10.1002/jcsm.12636 PMC7890271

[42] von Walden F , Liu C , Aurigemma N , Nader GA . mTOR signaling regulates myotube hypertrophy by modulating protein synthesis, rDNA transcription, and chromatin remodeling. Am J Physiol Cell Physiol 2016;311:663–672. 10.1152/ajpcell.00144.2016 27581648

[43] Fyfe JJ , Bishop DJ , Bartlett JD , Hanson ED , Anderson MJ , Garnham AP , et al. Enhanced skeletal muscle ribosome biogenesis, yet attenuated mTORC1 and ribosome biogenesis‐related signalling, following short‐term concurrent versus single‐mode resistance training. Sci Rep 2018;8:1–21. 29330460 10.1038/s41598-017-18887-6 PMC5766515

[44] Hammarström D , Øfsteng S , Koll L , Hanestadhaugen M , Hollan I , Apro W , et al. Benefits of higher resistance‐training volume are related to ribosome biogenesis. J Physiol 2020;598:543–565. 31813190 10.1113/JP278455

[45] Williamson D , Gallagher P , Harber M , Hollon C , Trappe S . Mitogen‐activated protein kinase (MAPK) pathway activation: effects of age and acute exercise on human skeletal muscle. J Physiol 2003;547:977–987. 12562918 10.1113/jphysiol.2002.036673 PMC2342728

[46] Zeng F , Zhao H , Liao J . Androgen interacts with exercise through the mTOR pathway to induce skeletal muscle hypertrophy. Biol Sport 2017;34:313–321. 29472733 10.5114/biolsport.2017.69818 PMC5819476

[47] Altamirano F , Oyarce C , Silva P , Toyos M , Wilson C , Lavandero González S , *et al*. Testosterone induces cardiomyocyte hypertrophy through mammalian target of rapamycin complex 1 pathway. 2009. 10.1677/JOE-09-0044 19474060

[48] Steiner JL , Fukuda DH , Rossetti ML , Hoffman JR , Gordon BS . Castration alters protein balance after high‐frequency muscle contraction. J Appl Physiol 2017;122:264–272. 27909227 10.1152/japplphysiol.00740.2016 PMC5338601

[49] Sato K , Iemitsu M . Exercise and sex steroid hormones in skeletal muscle. J Steroid Biochem Mol Biol 2015;145:200–205. 24704257 10.1016/j.jsbmb.2014.03.009

[50] Morton RW , Sato K , Gallaugher MP , Oikawa SY , McNicholas PD , Fujita S , et al. Muscle androgen receptor content but not systemic hormones is associated with resistance training‐induced skeletal muscle hypertrophy in healthy, young men. Front Physiol 2018;9:1373. 30356739 10.3389/fphys.2018.01373 PMC6189473

[51] Jiang M , Ma Y , Chen C , Fu X , Yang S , Li X , et al. Androgen‐responsive gene database: integrated knowledge on androgen‐responsive genes. Mol Endocrinol 2009;23:1927–1933. 19762544 10.1210/me.2009-0103 PMC5419166

[52] Hameed M , Orrell R , Cobbold M , Goldspink G , Harridge S . Expression of IGF‐I splice variants in young and old human skeletal muscle after high resistance exercise. J Physiol 2003;547:247–254. 12562960 10.1113/jphysiol.2002.032136 PMC2342624

[53] Wilkinson SB , Phillips SM , Atherton PJ , Patel R , Yarasheski KE , Tarnopolsky MA , et al. Differential effects of resistance and endurance exercise in the fed state on signalling molecule phosphorylation and protein synthesis in human muscle. J Physiol 2008;586:3701–3717. 18556367 10.1113/jphysiol.2008.153916 PMC2538832

[54] Marx JO , Ratamess NA , Nindl BC , Gotshalk LA , Volek JS , Dohi K , et al. Low‐volume circuit versus high‐volume periodized resistance training in women. Med Sci Sports Exerc 2001;33:635–643. 11283441 10.1097/00005768-200104000-00019

[55] von Haehling S , Morley JE , Coats AJ , Anker SD . Ethical guidelines for publishing in the Journal of Cachexia, Sarcopenia and Muscle: update 2019. J Cachexia Sarcopenia Muscle 2019;10:1143–1145. 31661195 10.1002/jcsm.12501 PMC6818444

