# Supplementary material for: Pharmacological hypogonadism impairs molecular transducers of exercise‐induced muscle growth in humans
Source: J Cachexia Sarcopenia Muscle. 2022 Mar 1;13(2):1134–50. doi: 10.1002/jcsm.12843 (PMC8977972; doi:10.1002/jcsm.12843)
Supplement: Supplementary file 1 — Figure S1 (related to Figure 2). Induced hypogonadism attenuates muscle growth and functional adaptations to RET. Values are means ± SEM. a = significantly different from baseline; b = significantly different between the two groups (Z: zoladex, P: placebo), P < 0.05. FFM: fat free mass, TFM: total fat mass, CSA: cross sectional area, MVC: maximal voluntary contraction, RET: resistance exercise training. Figure S2 (related to Figure 2). Muscle cross‐section stained for fibre type‐specific identification of satellite cells. Satellite cells are stained brown with Pax7 antibody, whereas laminin and type I fibres are stained with fluorescent green and type IIA fibres with fluorescent red. Myonuclei are stained blue (DAPI). Figure S3 (related to Figure 3). Hypogonadism attenuates muscle protein turnover increases in response to RET. Values are means ± SEM. b = significantly different between two groups (Z: zoladex, P: placebo), P < 0.05. FBR: fractional breakdown rate, RET: resistance exercise training. Figure S4 (related to Figure 4). Mechano‐signals cannot bypass blunted translational efficiency in hypogonadism after RET. Values are means ± SEM. a = significantly different from baseline; b = significantly different between the two groups (Z: zoladex, P: placebo), P < 0.05. NDUFB8: NADH dehydrogenase [ubiquinone] 1 beta subcomplex subunit 8, SDHB: Succinate dehydrogenase [ubiquinone] iron–sulfur subunit, UQCRC2: Cytochrome b‐c1 complex subunit 2, MTCO1: Mitochondrially encoded cytochrome c oxidase I, ATP5A: ATP synthase F1 subunit alpha, RET: resistance exercise training. Table S1. Primer sequences for each of the probed genes used in PCR. Table S2. Key resources. [file JCSM-13-1134-s001.docx]

**Supplementary Figures**


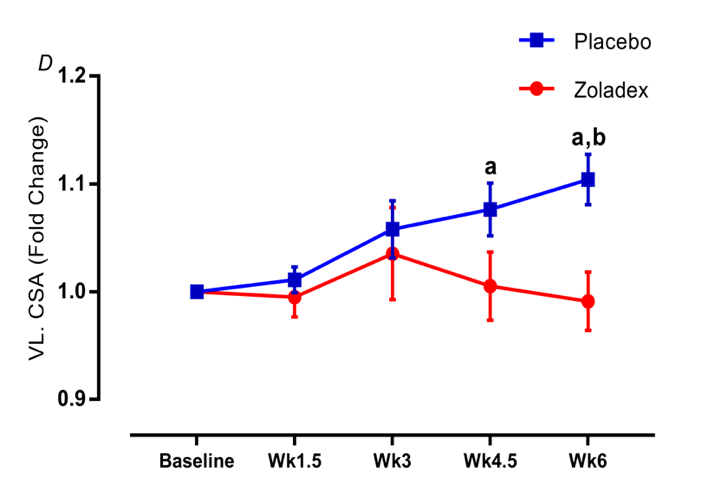

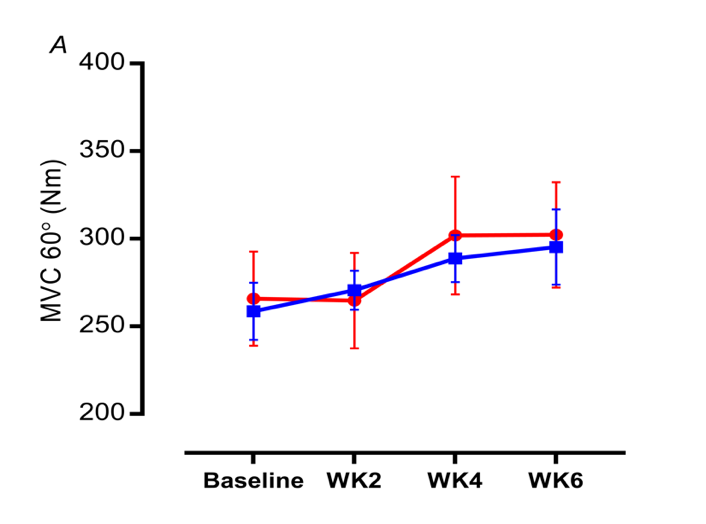

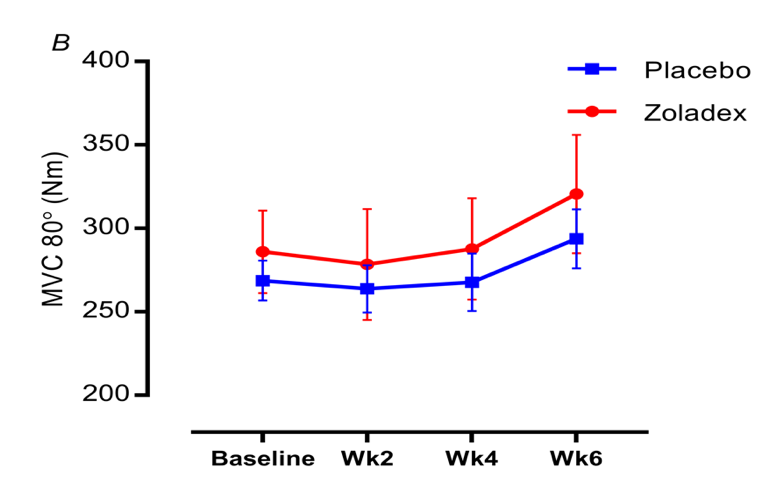


**Supplementary Figure 1 (related to Figure 2). Induced hypogonadism attenuates muscle growth and functional adaptations to RET.** Values are means ± SEM. **a** = significantly different from baseline; **b** = significantly different between the two groups (Z: zoladex, P: placebo), P<0.05. FFM: fat free mass, TFM: total fat mass, CSA: cross sectional area, MVC: maximal voluntary contraction, RET: resistance exercise training.


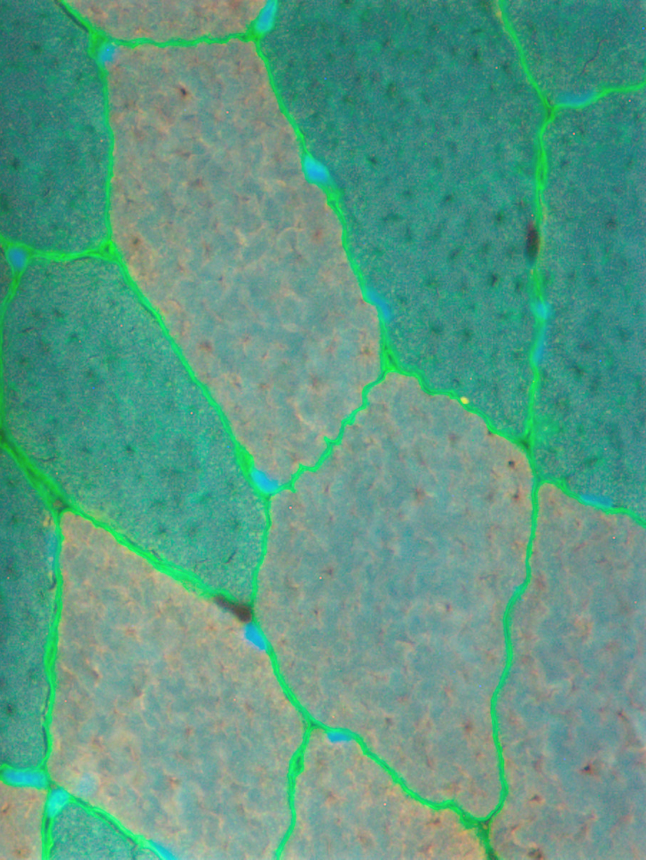


**Supplementary Figure 2 (related to Figure 2).** Muscle cross-section stained for fibre type-specific identification of satellite cells. Satellite cells are stained brown with Pax7 antibody, whereas laminin and type I fibres are stained with fluorescent green and type IIA fibres with fluorescent red. Myonuclei are stained blue (DAPI).

**Supplementary Figure 3 (related to Figure 3). Hypogonadism attenuates muscle protein turnover increases in response to RET.** Values are means ± SEM. **b** = significantly different between two groups (Z: zoladex, P: placebo), P<0.05. FBR: fractional breakdown rate, RET: resistance exercise training.

**Supplementary Figure 4 (related to Figure 4). Mechano-signals cannot bypass blunted translational efficiency in hypogonadism after RET.** Values are means ± SEM. **a** = significantly different from baseline; **b** = significantly different between the two groups (Z: zoladex, P: placebo), P<0.05. NDUFB8: NADH dehydrogenase [ubiquinone] 1 beta subcomplex subunit 8, SDHB: Succinate dehydrogenase [ubiquinone] iron-sulfur subunit, UQCRC2: Cytochrome b-c1 complex subunit 2, MTCO1: Mitochondrially encoded cytochrome c oxidase I, ATP5A: ATP synthase F1 subunit alpha, RET: resistance exercise training.

**Supplementary Tables**

| **Supplementary Table 1. Primer sequences for each of the probed genes used in PCR.** | | |
| --- | --- | --- |
|  | Forward | Reverse |
| RPL13A | 5’-TAAACAGGTACTGCTGGGCCG-3’ | 5’-CTCGGGAAGGGTTGGTGTTC-3’ |
| AR | 5’-GGTGAGCAGAGTGCCCTATC-3’ | 5’-GCAGTCTCCAAACGCATGTC-3’ |
| HSD17B2 | 5’-GGCTGGCATCTTATGGCTCA-3’ | 5’-CTGGTGCCTGCGATATTTGTT-3’ |
| HSD17B3 | 5’-TGTACTCAGCTTCCAAGGCG-3’ | 5’-TATGGGGTCAGCACCTGGAT-3’ |
| IGF-1Ea | 5’-TCAAATGTACTTCCTTCTGGGTC -3’ | 5’-TAAGGAGGCTGGAGATGTATTGC -3’ |
| IGF-1Ec | 5’-AAATCAGCAGTCTTCCAACCC-3’ | 5’-GTGTGCATCTTCACCTTCAAGAAA-3’ |
| Myogenin | 5’-CCAGGGGATCATCTGCTCACG-3’ | 5’-GGTTTCATCTGGGAAGGCCA-3’ |
| Myf-5 | 5’-GATGGCATGCCCGAATGTAAC-3’ | 5’-GCAATCCAAGCTGGATAAGGA-3’ |
| Myf-6 | 5’-CAAGAAAATCTTGAGGGTGCGG-3’ | 5’-TTAGCCGTTATCACGAGCCC-3’ |
| C-Myc | 5’-AACCACCACCATCCCTGTTTG-3’ | 5’-AAGGCCCCCAGACCCATTTC-3’ |
| MyoD | 5’-CTCCGACGGCATGATGGACTA-3’ | 5’-TGGGCGCCTCGTTGTAGTA-3’ |
| C-met | 5’-ACAGCTGACTTGCTGAGAGG-3’ | 5’-AGGTTTATCTTTCGGTGCCCA-3’ |
| PAX7 | 5’-CGGCCAGACTGCTGTTGATTAT-3’ | 5’-GAGTCCCAGCACAGCAGAGT-3’ |
| MSTN | 5’-TGGCTCAAACAACCTGAATCC-3’ | 5’-AAACGGATTCAGCCCATCTTCT-3’ |
| PGC1α | 5′-GAGTCATACTTGCTCTTGGTG-3′ | 5′-GATGATGGAGACAGCTATGGT-3′ |
| Tfam | 5′-TTCGTCCTCTTTAGCATGCTGA-3′ | 5′-CACCGCAGGAAAAGCTGAAG-3′ |

RPL13A: Ribosomal Protein L13A, AR: Androgen Receptor, HSD17B: Hydroxysteroid 17-Beta Dehydrogenase , IGF-1: insulin-like Growth Factor-1, MHC: Myosin Heavy Chain, Myf: Myogenic Factor, C-Myc: MYC Proto-Oncogene, BHLH Transcription Factor, MyoD: Myogenic Differentiation, C-met: MET Proto-Oncogene, Receptor Tyrosine Kinase, PAX7: Paired Box 7, MSTN: myostatin, PGC-1α: Peroxisome Proliferator-activated Receptor γ Co-activator-1α, Tfam: Mitochondrial Transcription Factor A.

| **Supplementary Table 2. Key resources** | | |
| --- | --- | --- |
| **REAGENT or RESOURCE** | **SOURCE** | **IDENTIFIER** |
| **Antibodies** | | |
| Rabbit AR | Cell Signaling Technology | Cat# 3202; RRID: AB_2060162 |
| Rabbit phospho-Akt^Ser473^ | Cell Signaling Technology | Cat# 9271; RRID: AB_329825 |
| Rabbit phosphor- mTOR^Ser2448^ | Cell Signaling Technology | Cat#2971; RRID: AB_330970 |
| Rabbit phospho-p70S6K^Thr389^ | Cell Signaling Technology | Cat#9234; RRID: AB_2269803 |
| Rabbit phospho-4E-BP1^Thr37/46^ | Cell Signaling Technology | Cat#2855; RRID: [AB_560835](http://antibodyregistry.org/AB_560835) |
| Rabbit phospho-AMPKα^Thr172^ | Cell Signaling Technology | Cat#2531; RRID: AB_330330 |
| Rabbit phospho-Raptor^Ser792^ | Cell Signaling Technology | Cat#2083; RRID: AB_2249475 |
| Rabbit phospho-tuberin/TSC2^Thr1462^ | Cell Signaling Technology | Cat#3617; RRID: AB_490956 |
| Rabbit phospho-FoxO3a^Ser253^ | Cell Signaling Technology | Cat#13129; RRID: AB_2687495 |
| Rodent OXPHOS | Abcam | Cat#ab110413; RRID: AB_2629281 |
| Mouse Pax7 | Developmental Studies Hybridoma Bank | RRID:AB_528428 |
| Mouse BA-F8 | Developmental Studies Hybridoma Bank | RRID:AB_10572253 |
| Mouse SC-71 | Developmental Studies Hybridoma Bank | RRID:AB_2147165 |
| Mouse 2E8 | Developmental Studies Hybridoma Bank | RRID:AB_2134060 |
| biotinylated goat antimouse secondary antibody | Vector Laboratories | Cat#PK6100 |
| Alexa Fluor 488 goat anti-mouse IgG | Thermo Fisher Scientific Invitrogen | Cat#A11001 |
| Alexa Fluor 488 goat anti-mouse IgG2b | Thermo Fisher Scientific Invitrogen | Cat#A21141 |
| Alexa Fluor 568 goat anti-mouse | Thermo Fisher Scientific Invitrogen | Cat#A11004 |
| **Chemicals, Peptides, Metabolites, and Recombinant Proteins** | | |
| Hydrochloric Acid (HCl) 37% | VWR | Cat#20252.335 |
| NH4OH | VWR | Cat#1336-21-6 |
| Dowex | Sigma-Aldrich | Cat#217506 |
| ETHANOL 100% | Fisher Scientific | Cat#6417-5 |
| XT MOPS | BioRad | Cat#1610788 |
| KCL | BDH | Cat#101985s |
| EGTA | Sigma-Aldrich | Cat#E3889 |
| Perchloric acid | BDH Laboratories | Cat#294587T |
| Sodium hydroxide | Sigma-Aldrich | Cat#30620 |
| Tris-HCL | Sigma-Aldrich | Cat#[1185-53-1](https://www.sigmaaldrich.com/catalog/search?term=1185-53-1&interface=CAS%20No.&N=0&mode=partialmax&lang=en&region=GB&focus=product) |
| EDTA | Sigma-Aldrich | Cat#E5134 |
| B-glycerophosphate | Sigma-Aldrich | Cat#154804-51-0 |
| Sodium fluoride | Sigma-Aldrich | Cat#450022 |
| ULTRA, EDTA-free Tablets, Protease Inhibitor | Sigma-Aldrich | Cat#5892791001 |
| Sodium orthovanadate | Sigma-Aldrich | Cat#S6508 |
| Bradford Reagent | Sigma-Aldrich | Cat#B6916 |
| Methanol 100% | Fisher Scientific | Cat#M/4056/17 |
| Methylchloroformate | Sigma-Aldrich | Cat#M35304 |
| Pyridine Anhydrous | Sigma-Aldrich | Cat#270970 |
| Chloroform | Sigma-Aldrich | Cat#C2432 |
| Sodium bicarbonate | BDH | Cat#27778.260 |
| Molecular Sieve | Sigma-Aldrich | Cat#334316 |
| Skimmed Milk Powder | Sigma-Aldrich | Cat#70166 |
| Bovine Serum Albumin | Sigma-Aldrich | Cat#A2153 |
| HRP Reagents | Millipore | Cat#WBKL S0 500 |
| Reprobe Stripping Buffer | Fisher Scientific | Cat#PN21059 |
| 20% Sodium Dodecyl Sulphate (SDS) | Fisher Scientific | Cat#S520053 |
| Glycerol | Sigma-Aldrich | Cat#G5516500 |
| 2-mercaptoethanol | Sigma-Aldrich | Cat#M7154 |
| Bromophenol Blue | Fisher Scientific | BP62044 |
| Tris Base | Fisher Scientific | Cat#BP152-1 |
| Glycine | Fisher Scientific | Cat#BP381-5 |
| Sodium Chloride (NaCl) | Sigma-Aldrich | Cat#S7653 |
| Tween-20 | Sigma-Aldrich | Cat#P9416 |
| RNase free water | Sigma-Aldrich | Cat#W4502 |
| Tri-reagent | Sigma-Aldrich | Cat#T9424 |
| Isopropanol | Sigma-Aldrich | Cat#I9030 |
| Vector Elite ABC horseradish peroxidase kit | Vector Laboratories | Cat#PK6100 |
| Immpact diaminobenzidine substrate | Vector Laboratories | Cat# SK-4105 |
| **Commercial Assays** | | |
| Testosterone ELISA | IBL International | Cat#RE52151 |
| High-Capacity cDNA Reverse Transcription Kit | Fisher Scientific | Cat#4374966 |
| 12% Criterion™ XT Bis-Tris Protein Gel | Biorad | Cat#3450119 |
| **Software** | | |
| ImageJ | NIH | https://imagej.nih.gov/ij/ |
| GraphPad Prism 7.01 | GraphPad Software | http://www.graphpad.com |
| Image Lab^TM^ 6.0.1 | Bio-Rad Laboratories | https://www.bio-rad.com |
| Nano Drop 2000/2000C 1.4.2 | Fisher Scientific | https://www.thermofisher.com |
| ISO-DAT 3.0 | Fisher Scientific | https://www.thermofisher.com |
| **Mitochondrial Extraction Buffer pH 7.5** | | |
| **Chemical** | **FW** | **Final Concentration** |
| MOPS | 209.3 | 20 mM |
| KCl | 74.55 | 110 mM |
| EGTA | 380.3 | 1 mM |
| **Homogenisation Buffer pH 7.5** | | |
| Tris-HCL | 157.6 | 50mM |
| EDTA | 372.2 | 1mM |
| EGTA | 380.4 | 1mM |
| Β-Glycerophosphate | 216.0 | 10mM |
| NaF | 41.99 | 50mM |
| **3x Laemmli Loading Dye (10 mls)** | | |
| 1M Tris-HCL pH 6.8 |  | 2.4ml |
| 20% Sodium dodecyl sulphate (SDS) |  | 3ml |
| Glycerol |  | 3ml |
| 2-Mercaptoethanol |  | 1.6ml |
| Bromophenol blue |  | 0.006g |
| **Western blot transfer buffer, Ph 8.3 (1 L)** | | |
| Tris base |  | 3.03g |
| Glycine |  | 14.4g |
| Methanol |  | 200ml |
| **Tris buffered saline with tween-20 (TBST), adjust to pH 7.6 (1 L)** | | |
| Sodium chloride (NaCl) |  | 8g |
| Tris base |  | 2.42g |
| Tween-20 |  | 1ml |
